# Supplementary material for: A phase II open label clinical study of the safety, tolerability and efficacy of ILB® for Amyotrophic Lateral Sclerosis
Source: PLoS One. 2022 May 25;17(5):e0267183. doi: 10.1371/journal.pone.0267183 (PMC9132272; doi:10.1371/journal.pone.0267183)
Supplement: S1 Appendix — (DOCX) [file pone.0267183.s001.docx]

|  | Clinical Study Protocol |  |
| --- | --- | --- |
|  | EudraCT No. | 2017-005065-47 |
|  | Investigational Medicinal Product | ILB |
|  | Study Code | TM-105 |
|  | Protocol Version and Date | Final version 1.5 2019-06-25 |

| Study title  A single-centre, open single-arm study where the safety, tolerability and efficacy of subcutaneously administered ILB will be evaluated in patients with Amyotrophic Lateral Sclerosis | | |
| --- | --- | --- |
| **Indication** | Amyotrophic Lateral Sclerosis (ALS) |  |
| **Development Phase** | IIa - pilot |  |
| **Design** | Open, single-arm |  |
| **Test treatment and dosage** | ILB (20 mg/ml) for subcutaneous injection |  |
| **Duration of treatment** | Weekly doses of 1 mg/kg, 5 doses in total |  |
| **Protocol signatories** | Lars Bruce, Sponsor’s Representative  Lennart Persson, MD, PhD, Principal Investigator |  |
|  |  |  |
|  |  |  |

*The following amendments have been made to the Final Clinical Study Protocol:*

| **Amendment No.** | **Date of Amendment** | **Revised protocol version** |
| --- | --- | --- |
| 1: In response to comments by MPA, Dnr 5.1-2018-21788 | 02 May 2018 | Version 1.1 |
| 2: Addition of Riluzole and Lamotrigine as prohibited concomitant medication, exclusion criteria and reason for permanent withdrawal. | 15 May 2018 | Version 1.2 |
| 3. Rephrasing of inclusion criterion 5 and exclusion criterion 9 to allow patients to fulfil these criteria before the study drug administration. | 23 August 2018 | Version 1.3 |
| 4. Replacing inclusion criterion 6 with exclusion criterion 12, to clarify that only patients with clinically significant abnormal PK-INR, fibrinogen, von Willebrand factor and APTT at screening should be excluded, and addition of potential interim analysis, | 17 December 2018 | Version 1.4 |
| 5. Administrative changes of the administrative structure and section added about pregnancy reporting | 25 June 2019 | Version 1.5 |

# STUDY SYNOPSIS

| **Study Title**  A single-centre, open single-arm study where the safety, tolerability and efficacy of subcutaneously administered ILB will be evaluated in patients with Amyotrophic Lateral Sclerosis | |
| --- | --- |
| **Study code**  TM-105 | **EudraCT No**  2017-005065-47 |
| **Study period**  Estimated date of first subject enrolled: Q2 2018  Estimated date of last subject completed: Q3 2019 | **Phase of development**  Phase IIa - pilot |
| **Study design and duration**  This is a Phase IIa single-centre, open single-arm study in patients with Amyotrophic Lateral Sclerosis.  Eligible subjects will be administered weekly doses of 1 mg/kg ILB. A total of 5 subcutaneous (s.c.) doses will be administered at the study clinic. The study consists of 10 visits; One 2-part screening visit, 5 IMP administration visits, and 3 follow-up visits. Each individual patient’s study participation will be approximately 4 months, including the screening and follow-up visits. | |
| **Number of patients planned**  15 patients | |
| **Objectives and endpoints**  Primary objective  The primary objective of the study is to evaluate the safety and tolerability of ILB in patients diagnosed with ALS.  *Primary endpoints*   - Frequency, seriousness and intensity of Treatment-emergent Adverse Events (TEAEs) - Physical examination - Vital signs - Electrocardiogram (ECG) recordings - Safety laboratory measurements: clinical chemistry, haematology including activated partial thromboplastin time (APTT)   Secondary objective  The secondary objective of the study is to evaluate efficacy of ILB in patients diagnosed with ALS.  *Secondary endpoints*   - Functional rating with ALS Functional Rating Scale – Revised (ALSFRS-R) - Functional rating with Norris scale - Evaluation of ALS biomarker Neurofilament Light chain (NFL) - Evaluation of biomarkers for neurological disorders (see 11.4.2) - Pulmonary function (FVC) - Quality of Life (QoL) - Autonomous symptoms - Levels of Hepatocyte Growth Factor (HGF) - Pharmacokinetic measurements of ILB in plasma - Changes in APTT (effect APTT)   Exploratory objectives (optional/to be decided)   - To evaluate the effect of ILB on levels of *e.g.* growth factors, cytokines and biomarkers in plasma and cerebrospinal fluid (CSF). | |
| **Safety and efficacy assessments**  Safety:   - AE assessments will start at administration of the first dose of ILB. - Physical exam will be obtained at all visits. On the days of ILB administration (Visit 2 to Visit 6), physical examination will be obtained before dosing. - Vital signs (blood pressure, heart rate and body temperature) will be obtained at all visits. - 12-lead ECG will be obtained at Visit 1 and Visit 7. - Lab tests (clinical chemistry, haematology, haemostatis) will be obtained at all visits. On the days of ILB administration (Visit 2 to Visit 6), lab tests will be obtained before dosing.   Efficacy:   - The investigator will complete the ALS Functional Rating Scale - Revised (ALSFRS-R) at all visits. On the days of ILB administration, rating scales will be completed before dosing. - The investigator will complete the Norris functional rating scales at all visits. On the days of ILB administration, rating scales will be completed before dosing. - Blood and cerebrospinal fluid (CSF) samples for evaluation of the ALS biomarker Neurofilament Light chain (NFL) will be obtained at Visit 1 after eligibility is confirmed and at Visit 7. - Blood and CSF samples for analysis of biomarkers for neurological disorders (see 11.4.2) will be obtained at Visit 1 after eligibility is confirmed and at Visit 7. - Respiratory function (FVC) will be evaluated at all visits. On the days of ILB administration (Visit 2 to Visit 6), respiratory function will be assessed before dosing. - Quality of Life (QoL) will be evaluated using Visual Analogue Scale (VAS)-based questionnaire filled out by patient and, if applicable, a next of kin, at Visit 1, Visit 2, Visit 4, Visit 6 and Visit 8. - Autonomous symptoms will be evaluated according to a 4-grade scale for a range of parameters at all visits. - Levels of Hepatocyte Growth Factor (HGF), pharmacokinetic measurements and effect APTT will be obtained in conjunction with the first and last ILB administration (Visit 2 and Visit 6). Blood samples will be obtained pre-dose, 30 minutes and 1, 2, 2.5, 3, 4, 6 hours after dosing. | |
| **Eligibility criteria**  Inclusion criteria:   1. Willing and able to give written informed consent for participation in the study. 2. Clinical diagnosis of Amyotrophic Lateral Sclerosis. 3. Male or female patients between 18 to 80 years of age (inclusive). 4. Forced Vital Capacity (FVC) ≥ 65% of predicted value for gender, height and age at screening. 5. Evaluated with ALSFRS-R and Norris clinical rating scales for at least the past 4 weeks before study drug administration.   Exclusion criteria:   1. Unable to understand information about the study or are expected not to collaborate with the study team. 2. Concurrent serious disease, other than ALS, at the discretion of the Investigator. 3. Pregnancy. 4. Patients of child-bearing potential not willing to use adequate double contraception with < 1% failure rate after the screening visit until the last visit. 5. Addiction to drugs or alcohol. 6. Confirmed HIV, Hepatitis B or C. 7. Known bleeding disorders or abnormal bleeding events. 8. Treatment with anticoagulant drugs warfarin and novel oral anticoagulants (NOAC) within the last 14 days prior to screening. 9. Treatment with Riluzole or Lamotrigine within the last 28 days prior to study drug administration. 10. Hypersensitivity to dextran sulfate. 11. Poor venous access. 12. Patients with clinically significant abnormal PK-INR, fibrinogen, von Willebrand factor and activated partial thromboplastin time (APTT) at screening. | |
| **Investigational Medicinal Product (IMP), dosage and mode of administration**  The active pharmaceutical ingredient of the investigational medicinal product (IMP) is dextran sodium sulfate salt, 5 kDa, 20 % sulfatation (ILB). ILB is a solution for subcutaneous (s.c.) injection and consists of 20 mg/ml ILB in 0.9% NaCl. Each glass vial contains 10 ml.  The dose administered will depend on the subject’s body weight at the second study visit, prior to the first ILB administration. ILB will be injected s.c. on alternating sides of the abdomen, thigh, or the buttock (in that order of priority). | |
| **Statistical methods**  No formal sample size calculation has been performed for this study. The proposed sample size is considered sufficient in this early phase II development to provide adequate information on the patients.  Categorical data will be presented as counts and percentages. Individual patient data will be listed by subject number and, where applicable, assessment time.  Continuous data will be summarised using descriptive statistics. The number of observations, mean, median, maximum, minimum and standard deviation will be reported. Individual patient data will be listed by subject number and, where applicable, assessment time.  Individual plasma concentration-time data of ILB will be evaluated using a validated Phoenix WinNonlin® software. The PK parameters will be calculated by non-compartmental analysis (NCA). Descriptive statistics for the assessed PK parameters will be presented for each parameter and first and last ILB administration (Visit 2 and Visit 6), as well as pooled data over the dosing days (if appropriate).  A more technical and detailed elaboration of the principal features will be presented in a separate Statistical Analysis Plan (SAP). | |

# TABLE OF CONTENTS

[2 STUDY SYNOPSIS 3](#_Toc532822684)

[3 TABLE OF CONTENTS 7](#_Toc532822685)

[4 LIST OF ABBREVIATIONS 11](#_Toc532822686)

[5 Investigator and study administrative structure 13](#_Toc532822687)

[6 INTRODUCTION 15](#_Toc532822688)

[6.1 Investigational background 15](#_Toc532822689)

[6.2 Investigational product ILB 16](#_Toc532822690)

[6.3 Rationale for use of ILB in ALS 16](#_Toc532822691)

[6.4 Risk and benefit assessment 17](#_Toc532822692)

[7 STUDY OBJECTIVES AND ENDPOINTS 20](#_Toc532822693)

[7.1 Primary objective 20](#_Toc532822694)

[7.2 Secondary objectives 20](#_Toc532822695)

[7.3 Exploratory objectives 20](#_Toc532822696)

[8 INVESTIGATIONAL PLAN 21](#_Toc532822697)

[8.1 Study design 21](#_Toc532822698)

[8.2 Rationale for study design and dose 21](#_Toc532822699)

[8.3 Overall study procedures 21](#_Toc532822700)

[9 STUDY POPULATION 26](#_Toc532822701)

[9.1 Screening log 26](#_Toc532822702)

[9.2 Number of subjects 26](#_Toc532822703)

[9.3 Inclusion criteria 26](#_Toc532822704)

[9.4 Exclusion criteria 26](#_Toc532822705)

[9.5 Prior and concomitant therapy 27](#_Toc532822706)

[9.6 Withdrawal of patients from therapy or assessment 27](#_Toc532822707)

[10 TREATMENT 29](#_Toc532822708)

[10.1 Treatment administration 29](#_Toc532822709)

[10.2 Identity of Investigational Medicinal Product 29](#_Toc532822710)

[10.3 Packaging and labelling 29](#_Toc532822711)

[10.4 Conditions for storage 30](#_Toc532822712)

[10.5 Dispensing and accountability 30](#_Toc532822713)

[10.6 Treatment compliance 30](#_Toc532822714)

[11 STUDY ASSESSMENTS 31](#_Toc532822715)

[11.1 Recording of data and time windows 31](#_Toc532822716)

[11.2 Demographics and other baseline characteristics 31](#_Toc532822717)

[11.3 Assessments related to the primary endpoint 32](#_Toc532822718)

[11.4 Assessments related to secondary endpoints 34](#_Toc532822719)

[11.5 Assessments related to the explorative objectives 36](#_Toc532822720)

[11.6 Total volume of blood and CSF per patient 36](#_Toc532822721)

[11.7 Biobank 36](#_Toc532822722)

[12 Safety 37](#_Toc532822723)

[12.1 Expected effects 37](#_Toc532822724)

[12.2 Definitions 37](#_Toc532822725)

[12.3 Reporting 39](#_Toc532822726)

[13 ETHICAL AND REGULATORY REQUIREMENTS 41](#_Toc532822727)

[13.1 Ethical conduct of the study 41](#_Toc532822728)

[13.2 Ethics and regulatory review 41](#_Toc532822729)

[13.3 Subject information and consent 41](#_Toc532822730)

[13.4 Subject data protection 42](#_Toc532822731)

[13.5 Protocol deviations and amendments 42](#_Toc532822732)

[14 Quality control and quality assurance 43](#_Toc532822733)

[14.1 Training of study site personnel 43](#_Toc532822734)

[14.2 Clinical Monitoring 43](#_Toc532822735)

[14.3 Audits and inspections 43](#_Toc532822736)

[15 DATA MANAGEMENT 43](#_Toc532822737)

[15.1 Source data 43](#_Toc532822738)

[15.2 Case Report Form 44](#_Toc532822739)

[15.3 Data Management Plan 44](#_Toc532822740)

[15.4 External Data 44](#_Toc532822741)

[15.5 Archiving 44](#_Toc532822742)

[16 STATISTICAL METHODS AND DETERMINATION OF SAMPLE SIZE 45](#_Toc532822743)

[16.1 General 45](#_Toc532822744)

[16.2 Sample size determination 45](#_Toc532822745)

[16.3 Analysis data sets 45](#_Toc532822746)

[16.4 Description of study population 45](#_Toc532822747)

[16.5 Analysis of primary endpoints 46](#_Toc532822748)

[16.6 Efficacy endpoints 46](#_Toc532822749)

[16.7 Statistical/analytical issues 47](#_Toc532822750)

[17 insurance and financing 48](#_Toc532822751)

[17.1 Insurance 48](#_Toc532822752)

[17.2 Financing 48](#_Toc532822753)

[18 Study time table and discontinuation 48](#_Toc532822754)

[19 Publication policy 49](#_Toc532822755)

[20 APPENDIX 50](#_Toc532822756)

[20.1 Appendix 1. Risk management 50](#_Toc532822757)

[20.2 Appendix 2: ALSFRS-R Rating Scale 52](#_Toc532822758)

[20.3 Appendix 3: Norris Rating Scale 55](#_Toc532822759)

[20.4 Appendix 4: Livskvalitet - försöksperson 57](#_Toc532822760)

[20.5 Appendix 5: Livskvalitet - närstående 58](#_Toc532822761)

[20.6 Appendix 6: Autonoma och sensoriska symtom 59](#_Toc532822762)

[21 Study protocol agreement 60](#_Toc532822763)

[22 References 61](#_Toc532822764)

**List of Tables**

[Table 1 Overview of clinical studies with ILB 18](#_Toc532822765)

[Table 2 Schedule of events 22](#_Toc532822766)

[Table 3 Detailed schedule of events for Visit 2 and Visit 6 24](#_Toc532822767)

[Table 4 Detailed schedule of events for Visit 4 25](#_Toc532822768)

**List of Figures**

[Figure 1 Chemical structure of ILB 16](file:///T:\TX%20Medic%20AB\Research%20&%20Development\Studies\3_Clinical\Phase%20II\TM-105%20ALS%20Sahlgrenska\CSP\Version%201.4\TM-105%20CSP%20final%201.4%2020181217_TC.docx#_Toc532822769)

[Figure 2 Information on IMP vial label 30](#_Toc532822770)

[Figure 3 Information on IMP box label 30](#_Toc532822771)

# LIST OF ABBREVIATIONS

| AE | Adverse Event |
| --- | --- |
| ALS | Amyotrophic Lateral Sclerosis |
| ALSFRS-R | ALS Functional Rating Scale - Revised |
| API | Active Pharmaceutical Ingredient |
| APL | Apoteket Produktion & Laboratorier |
| APTT | Activated Partial Thromboplastin Time |
| AUC | Area Under the plasma concentration - time Curve |
| AUC_0-t_ | area under the curve from 0 to t hours |
| AUC_inf_ | AUC from timepoint 0 until infinity |
| AUC_last_ | AUC from timepoint 0 until the last timepoint t |
| AUCtau | AUC in a dosing interval |
| BDNF | Brain Derived Neurotrophic Factor |
| BP | Blood Pressure |
| CA | Competent Authority |
| C_max_ | maximum observed plasma concentration |
| CRF | Case Report Form |
| CSF | Cerebrospinal Fluid |
| EC | Ethics Committee |
| ECG | Electro Cardio Gram |
| ELISA | Enzyme-Linked immunosorbent Assay |
| FGF | Fibroblast Growth Factor |
| FVC | Forced Vital Capacity |
| GCP | Good Clinical Practice |
| HGF | Hepatocyte Growth Factor |
| HIV | Human Immunodeficiency Virus |
| HUVEC | Human Umbilical cord Vascular Endothelial Cell |
| i.v. | intravenous |
| IB | Investigator's Brochure |
| ICF | Informed Consent Form |
| ICH | International Conference on Harmonization |
| ILB | Study drug / investigational medicinal product |
| IMP | Investigational Medicinal Product |
| kDa | kilo Dalton |
| LMW-DS | Low Molecular Weight Dextran Sulfate |
| Mw | Molecular Weight |
| PK | Pharmacokinetic |
| PK-INR | Prothrombin Complex International Normalised Ratio |
| PT | Preferred Term |
| QoL | Quality of Life |
| s.c. | subcutaneous |
| SAE | Serious Adverse Event |
| SAP | Statistical Analysis Plan |
| SDV | Source Data Verification |
| SOC | System Organ Class |
| SU | Sahlgrenska University Hospital |
| SUSAR | Suspected Unexpected Serious Adverse Reaction |
| t_1/2_ | half-life |
| TEAE | Treatment-Emergent Adverse Events |
| t_last_ | time at which the last plasma concentration was observed |
| t_max_ | Time at which Cmax was observed |
| VAS | Visual Analogue Scale |
| VEGF | Vascular Endothelial Growth Factor |
| WOCBP | Women of childbearing potential |

# Investigator and study administrative structure

| Principal investigator  Name: Lennart Persson, MD, PhD  Address: Section of Clinical Neurosciences  Sahlgrenska University Hospital  413 45 Göteborg  Phone: 070-794 59 43  E-mail: lennartpersson@msn.com |  |
| --- | --- |
| SPONSOR  TikoMed AB  Name: Lars Bruce  Address: Box 81  263 03 Viken  Phone: 042-23 84 44  Fax: 042-23 84 91  E-mail: florence.lange@tikomed.com | SPONSOR’S MEDICAL EXPERT  Name: Anders Svensson, MD, PhD  Address: Högadalsgatan 2B  431 69 Göteborg  Phone: 0708-92 92 91  E-mail: anders.svensson@tikomed.com |
| Clinical REsearch Organisation (CRO):  Link Medical Research AB  Project Manager Name: Lina Hallberg  Address: Jörgen Kocksgata 4  211 20 Malmö  Phone: +46 73 025 04 41    E-mail: lina.hallberg@linkmedical.eu |  |
|  |  |
| Trial site  CTC Clinical Trial Center  Name: Christer Söderström  Address: Sahlgrenska University Hospital  413 45 Göteborg  Phone: 070-082 50 90  E-mail: christer.soderstrom@vgregion.se |  |
|  |  |
| BIOSTATISTICIAN  CTC AB  Name: Fredrik Hansson  Address: Dag Hammarskjöldsväg 13  752 37 Uppsala  Phone: 018-30 33 00  Email: fredrik.hansson@clinicaltrialconsultants.se | Pharmacokineticist  PKxpert AB  Name: Annsofi Nihlén  Address: Tomtebogatan 32  113 38 Stockholm  Phone: 0765-69 81 75  E-mail: annsofi.nihlen@pkxpert.com |
|  |  |
|  |  |
| Laboratory (clinical chemistry)  Name: Susanne Iversen-Hemberg  Address: Sahlgrenska University Hospital  413 45 Göteborg  Phone: 031-342 1000 | Laboratory (Bacteriology)  Name: Stefan Lange  Address: Sahlgrenska University Hospital  413 45 Göteborg  Phone: 031-342 1000 |
| Laboratory (Neurochemistry)  Name: Kaj Blennow  Address: Mölndal University Hospital  431 80 Göteborg  Phone: 031-342 1000  Laboratory (Clinical immunology)  Name: Anna Lundgren  Address: Sahlgrenska University Hospital  413 45 Göteborg  Phone: 0700 20 67 70  e-mail: anna.lundgren@vgregion.se | Laboratory (Pharmacokinetics)  Eurofins BioPharma Product Testing Munich GmbH  Name: Dr Felicia Kirschner  Address: Behringstr. 6-8, 82152 Planegg, Germany  Phone: +49898996500  e-mail: [feliciakirschner@eurofins.com](mailto:feliciakirschner@eurofins.com) |
|  |  |

# INTRODUCTION

## Investigational background

Amyotrophic lateral sclerosis (ALS, also known as Lou Gehrig’s disease) is the most common motor neuron disease. It is a fatal disease, affecting most populations of neurons. The most important degeneration of neuronal cells occurs in motor neurons in spinal cord, brain stem and brain. The disease begins focally in the central nervous system and then spreads relentlessly[1]. The clinical diagnosis, defined by progressive signs and symptoms of upper and lower motor neuron dysfunction, is confirmed by clinical findings, electromyography, blood and cerebrospinal fluid (CSF) analysis. Additional testing with for instance Magnetic Resonance Imaging (MRI) excludes other conditions. The rate of progress of the disease is related to neuronal markers in CSF and blood. CSF levels of Neurofilament light chain (NFL) is the most important indicator of the activity of the disease [2-4]. The disease is heterogeneous, but most patients die of respiratory muscle weakness less than 3-5 years from symptom-onset. Like other age-related neurodegenerative diseases, ALS has genetic, metabolic and environmental triggers.

There is yet no cure for ALS, and management is focused on a combination of neuroprotective medication, multidisciplinary clinics and respiratory support. Speciality clinics for ALS emerged in the 1980s and most large centres in developed countries currently offer multidisciplinary care [5]. The disease causes severe decrease of the quality of life in both patients and next of kin [6-8]. Patients treated by ALS care teams may have higher quality of life [9] and longer survival [10]. Malnutrition and dehydration are common as ALS advances. Later therapy often includes percutaneous gastric feeding and non-invasive ventilatory support, which increases time of survival and increased quality of life of both patient and next of kin.

To date, one medication (riluzole, licensed in 1996) has been proved to prolong survival somewhat in ALS [11]. Riluzole was developed because it possesses anti-glutamatergic properties that might reduce excitotoxicity in ALS. Riluzole slowed disease progression in two randomized controlled trials [12, 13], delaying death with weeks or a few months [11]. The most common side effects are exanthema, diarrhoea, dizziness, fatigue, nausea, and somnolence. Elevation of liver enzymes can occur, but rarely to levels that are clinically meaningful. Many patients in Europe and more than half of ALS patients in the U.S. take riluzole [14].

Numerous trials have so far been unable to identify another neuroprotective agent. Researchers now aim to slow disease progression by targeting known pathophysiological pathways or genetic defects. Until better understanding of the causes and mechanisms underlying progression that lead to more robust neuroprotective agents, symptomatic therapies can extend life and improve quality of life. Palliative care programs such as hospice give emotional and physical support to patients and families throughout much of the disease course [15].

In this Phase II pilot study in ALS patients, safety and tolerability of ILB will be evaluated after repeated subcutaneous (s.c.) doses of ILB (1 mg/kg, 5 doses in total) during 4 weeks with 10-week follow-up. The secondary objective is to investigate efficacy through documentation of functional activity as assessed by clinical rating scales, respiratory function and biological markers.

## Investigational product ILB

The Active Pharmaceutical Ingredient (API) ILB is a type of low molecular weight dextran sulfate (LMW-DS). ILB is a polyanionic derivative of dextran which is a polymer of anhydroglucose which contains approximately 20 % sulfur (Figure 1). Each glucose unit in the dextran chain has approximately two sulfate groups, normally located at the second and fourth carbon of the glucose units.

Preparation of ILB is accomplished by alcohol fractionation, in which the largest molecular weight dextran sulfate precipitates first. The final product has an average molecular weight (Mw) of 5 kDa and contains molecules spanning approximately 3 - 8 kDa. It is supplied as the sodium salt which is a white to off-white powder freely soluble in water and salt solutions (> 100 mg/ml).


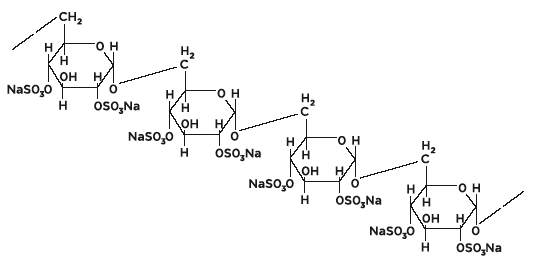

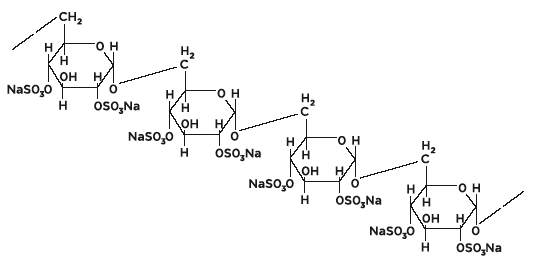


…

Figure 1 Chemical structure of ILB

During GMP manufacture of the Investigational Medicinal Product (IMP) ILB, the API is dissolved in sterile water with 9 mg/ml NaCl and filter sterilised to a clear solution.

## Rationale for use of ILB in ALS

HGF is a potent survival-promoting factor for motor neurons and has been suggested for ALS treatment [16, 17]. Human recombinant HGF (hrHGF) has been shown to attenuate spinal motor neuron degeneration in transgenic ALS rat models, and improve functional recovery in a non-human primate model of contusive cervical spinal cord injury [18, 19]. In a model of ALS in mice (SOD1 mutation), over-expression of HGF in the nervous system showed reduced motor neuron death and axonal degeneration as well as a prolonged life span [20]. HGF appears to be a good candidate for the treatment of ALS since it passes the blood-brain barrier (BBB) [21] and the HGF levels in ALS patients seems dysregulated [22]. ILB administration increases the plasma level of HGF in human healthy volunteers and in rats and mice [23].

Other growth factors with potential therapeutic use in ALS are vascular endothelial growth factor (VEGF) and fibroblast growth factors (FGF) [24]. VEGF treatment increases survival, delay disease onset, improve motor functions, protect motor neurons and the neuromuscular junction in studies on rodent ALS models. VEGF is an attractive therapeutic factor since it passes the blood brain barrier and is down-regulated in the late stage of ALS. Fibroblast Growth Factor (FGF) can protect motor neurons after axotomy-induced apoptosis and support neuronal regeneration. Sulfated polysaccharides, like ILB, have been shown to bind and affect levels and potentiate VEGF, BDNF and FGF signalling/effects [25].

In conclusion, based on nonclinical data it is possible that a positive effect of ILB in ALS would be mediated through an increase of tissue growth actors.

## Risk and benefit assessment

So far, five clinical studies have been conducted with ILB administered intravenously (i.v.) or subcutaneously (s.c.) in 79 subjects in doses up to 24 mg/kg; four studies in healthy volunteers (n=69) and one Phase-II study in diabetic patients (n=10) in association with standard of care cell therapy treatment (Table 1). In all clinical studies, there were an expected dose-dependent prolongation of activated partial thromboplastin time (APTT), up to about 1100 seconds after 24 mg/kg i.v. Increased numbers of lymphocytes, white blood cells and especially a dose-dependent increase of HGF were recorded in the studies.

Table 1 Overview of clinical studies with ILB

| **Subjects (n)** | **Substance** | **Dose**  **(mg/kg)** | **Administration** | **Adverse Events** | **Study phase,**  **Name,**  **Eudract no.** |
| --- | --- | --- | --- | --- | --- |
| Healthy subjects (34) | ILB  Heparin as control | 4.5-15.7 | *i.v.* bolus (1.5 mg/kg) +  20 min (3 mg/kg) infusion + 5 h (~9 mg/kg) infusion | Well-tolerated. Two subjects reported  hypersensitivity  reaction. | Phase I,  LMW-DS-101, 2006-005193-42 |
| Diabetes Type 1 patients (10) | ILB  Heparin as control | Up to 18.3 | *i.v.* Bolus (1.5 mg/kg) + 20 min (3 mg/kg) infusion + 5 h (max 13.8 mg/kg) infusion | No SAEs related to ILB. ILB is as safe and effective as heparin standard therapy. | Phase II study,  CIT-01^1^, 2008-001210-25 |
| Healthy subjects (24) | ILB | 3-24 | 10 min *i.v.* infusion | All doses including 24 mg/kg are evaluated as safe and well tolerated. | Phase I/II,  LMW-DS-102, 2011-004111-23 |
| Healthy subjects (3) | ILB    G-CSF (filgrastim) | 18  0.01 | 10 min *i.v*. infusion  *s.c.* injection, once daily for 5 days | ILB 18 mg/kg in combination with filgrastim treatment is evaluated as safe and well tolerated. | Phase I/II,  LMW-DS-103, 2014-000659-10 |
| Healthy subjects (12) | LMW-DS,  ILB  (5 kDa) | 6 | Single *s.c.* injection +  Single *i.v.* injection | s.c. administration of ILB 100 mg/ml is considered safe, and with approx. 88 % bioavailability. | Phase I,  TM-104  2016-004468-20 |

^1^ TikoMed AB was IMP supplier. The study was sponsored by the National Institute of Health (NIH, USA).

Specific risks related to the treatment with ILB in this study are local injection-site reactions and hypersensitivity reactions:

In a clinical trial, two healthy volunteers reported mild and transient hypersensitivity reactions, which was judged to have a causal relationship with ILB administration. No actions were taken to treat the events, since the reactions stopped spontaneously. Forty-two healthy subjects have been dosed with approximately 6-16 mg/kg ILB without concomitant treatment with antihistamines, out of which two showed hypersensitivity reactions. As the hypersensitivity reaction was mild and transient, and occurred in only two subjects, treatment with antihistamines will not be included in this study.

The local tolerance of s.c. administration was evaluated in a phase I study in 12 healthy volunteers receiving 2-3 s.c. injections (each approx. 2 ml) of 100 mg/ml ILB in the abdomen (6 mg/kg). The local tolerability showed mild effects after 10 minutes in terms of pain, pruritus, erythema and oedema. The effects were transient and had normalised at 30 minutes or 4 hours after the injection.

In toxicology studies in rats, it has been shown that LMW-DS compounds – including ILB – had the potential to induce lung toxicity and respiratory dysfunction. To address this issue, a number of complementary toxicological studies have been performed in rats, mice and monkeys, and lung function has been studied carefully in the clinical studies with ILB. The conclusion is that the effects observed on the lungs in rat seem to be species related, as it is not seen in the nonclinical studies in mice or monkeys, and neither in previous clinical studies. However, due to the lung findings in the rat, the lung function will be monitored in the planned clinical study with intermittent forced vital capacity (FVC) respiratory tests.

Toxicology studies have been performed in both male and female rats and monkeys but as there are no data available at this stage on reproductive toxicology, all included study subjects – and their partner – of childbearing potential must use adequate double contraceptive protection during the study (Visit 2 to Visit 9). A woman is considered of childbearing potential (WOCBP) i.e. fertile, following menarche and until becoming post-menopausal – as defined as no menses for at least 12 months without alternative medical cause – or permanently sterile through hysterectomy, bilateral salpingectomy and bilateral oophorectomy. A man is considered fertile after puberty unless permanently sterile by bilateral orchidectomy.

ILB prolongs APTT in a dose-dependent manner, and after prolonged treatment in humans with a similar compound, but with higher molecular weight, thrombocytopenia has been observed. Both these effects might lead to bleeding complications. Despite this, provoked (IVY method) or spontaneous bleedings have not been observed in any of the hitherto performed clinical studies with ILB and are not expected to occur in this study, except possibly at the injection site.

ALS is a terminal disease where most patients die of respiratory muscle weakness less than 3-5 years from symptom onset. As there is yet no cure for ALS, and only one drug on the market that slightly prolongs the survival, there is for ALS patients a potential major benefit for developing a drug that may slow or reverse the disease progression.

Taken together, the data from nonclinical toxicity studies performed with ILB in mice, rats and monkey, data from studies in healthy subjects and diabetes patients, and additional information obtained from the literature with similar substances, indicate that the proposed dose and administration route in the protocol can be used in the present investigation without safety concerns.

For more detailed information about the toxicological and clinical data, refer to the Investigator’s Brochure (IB).

# STUDY OBJECTIVES AND ENDPOINTS

## Primary objective

The primary objective of the study is to evaluate the safety and tolerability of ILB in patients diagnosed with ALS.

### Primary endpoint

- Frequency, seriousness and intensity of Treatment-emergent Adverse Events (TEAEs)
- Physical examination
- Vital signs
- Electrocardiogram (ECG) recordings
- Safety laboratory measurements: clinical chemistry, haematology including activated partial thromboplastin time (APTT)

## Secondary objectives

The secondary objective of the study is to evaluate efficacy of ILB in patients diagnosed with ALS.

### Secondary endpoints

- Functional rating with ALS Functional Rating Scale – Revised (ALSFRS-R)
- Functional rating with Norris scale
- Evaluation of ALS biomarker Neurofilament Light chain (NFL)
- Evaluation of biomarkers for neurological disorders (see 11.4.2)
- Pulmonary function (FVC)
- Quality of Life (QoL)
- Autonomous symptoms
- Levels of Hepatocyte Growth Factor (HGF)
- Pharmacokinetic measurements of ILB in plasma
- Changes in APTT (effect APTT)

## Exploratory objectives

To evaluate the effect of ILB on levels of *e.g.* growth factors, cytokines and biomarkers in plasma and cerebrospinal fluid (CSF).

# INVESTIGATIONAL PLAN

## Study design

This is a single-centre, open single-arm study where the safety, tolerability and efficacy of subcutaneously (s.c.) administered ILB will be evaluated in patients with Amyotrophic Lateral Sclerosis (ALS). There will be 10 visits to the clinic: 1 2-part screening visit (Visit 1a and Visit 1b), 5 IMP administration visits (Visit 2 to Visit 6) and 3 follow-up visits (Visit 7 to Visit 9). Each individual patient’s study participation will be approximately 4 months, including the screening and follow-up visits.

## Rationale for study design and dose

The design of the study is based on the aim to study safety, tolerability and efficacy of ILB solution in patients with ALS. Doses up to 24 mg/kg ILB has been administered to 79 healthy volunteers, but as ILB has never been administered to ALS patients and never more than two subsequent injections, a careful approach with low (1 mg/kg) and few doses (5 injections) with 1-week dosing interval has been chosen. The combined safety data from nonclinical and clinical studies indicate that the proposed dose can be administered without any safety concerns.

No control group is used as there are no spontaneous remissions or recovery of the disease. The progression of the disease is linear in the two clinical rating scales used, and therefore the rating of disease progression is reliable and standard in ALS research.

## Overall study procedures

Subjects will be screened for eligibility according to pre-defined criteria (see section 9.3 and 9.4) within 30 days prior to first administration of IMP (Visit 1a; day -30 to -7). If eligibility is confirmed at Visit 1a, the patients will return at Visit 1b (day -23 to -1) for collection of CSF and blood for analysis of NFL and other biomarkers of neurological disorders (see 11.4.2), and – if consented by patient – exploratory purposes (see 11.5). If it is found to be appropriate by patient or Investigator, the screening tests can be performed over several visits to avoid too strenuous strain on patient and next of kin.

Each subject will receive oral and written information about the study and the planned investigations and will have to give his or her signed and dated informed consent before any study-related procedures are initiated. On recruitment to the study, the patients will be evaluated for inclusion and exclusion criteria, and undergo a baseline assessment to include demographics, medical history, concomitant medication, vital signs, physical examination (including weight and height), ECG, haematology, and clinical chemistry. Patients will also complete a baseline assessment on the ALS Functional Rating Scale (ALSFRS-R), Norris rating scale and a quality of life assessment (Appendix 2-5). The patients will during 5 visits be administered ILB 1 mg/kg per week (Visit 2 to Visit 6), followed by 3 follow up visits (Visit 7 to Visit 9) over 10 weeks after the last dose. During the clinical trial, the patients will undergo regular assessments for safety and efficacy parameters.

The overall schedule of events for the study is presented in Table 2. For the visits with critical time points for study assessments, detailed schedule of events are presented in Table 3 (Visit 2 and Visit 6) and Table 4 (Visit 4). The procedures for all study assessments are described in detail in section 11.

Table 2 Schedule of events

| **Visit** | **1a** | **1b** | **2** | **3** | **4** | **5** | **6** | **7** | **8** | **9** |
| --- | --- | --- | --- | --- | --- | --- | --- | --- | --- | --- |
|  | Screening | Screening | Dosing^7^ | Dosing^7^ | Dosing^7^ | Dosing^7^ | Dosing^7^ | Follow-up | Follow-up | End of study  follow-up |
| **Day** | -30 to -7 | -23 to -1 | 1 | 8 | 15 | 22 | 29 | 36 | 50 | 99 |
| **Time window (days)** |  |  |  | ± 3 | ± 3 | ± 3 | ± 3 | ± 3 | ± 7 | ± 7 |
| Informed consent | X |  |  |  |  |  |  |  |  |  |
| Information on contraception | X |  |  |  |  |  |  |  |  |  |
| Information on medication | X |  |  |  |  |  |  |  |  |  |
| Eligibility criteria | X |  |  |  |  |  |  |  |  |  |
| Demographics | X |  |  |  |  |  |  |  |  |  |
| Medical history | X |  |  |  |  |  |  |  |  |  |
| Concomitant medications | X | X | X | X | X | X | X | X | X | X |
| Physical examination | X |  | X | X | X | X | X | X | X | X |
| Weight | X |  | X |  |  |  |  | X |  |  |
| Height | X |  |  |  |  |  |  |  |  |  |
| Vital signs | X |  | X | X | X | X | X | X | X | X |
| Forced vital capacity (FVC) | X |  | X | X | X | X | X | X | X | X |
| Haematology, including PK-INR | X |  | X | X | X | X | X | X | X | X |
| Clinical chemistry | X |  | X | X | X | X | X | X | X | X |
| Pregnancy^1^ | X |  | X | X | X | X | X | X | X | X |
| Drugs of abuse | X |  |  |  |  |  |  |  |  |  |
| Gamma-glutamyltransferase | X |  |  |  |  |  |  |  |  |  |
| HIV, hepatitis B and C | X |  |  |  |  |  |  |  |  |  |
| ECG | X |  |  |  |  |  |  | X |  |  |
| ALSFRS-R and Norris rating scales | X |  | X | X | X | X | X | X | X | X |
| Quality of life assessment | X |  | X |  | X |  | X |  | X |  |
| Autonomous symptoms | X |  | X | X | X | X | X | X | X | X |
| Biomarkers (CSF, plasma, serum) |  | X |  |  |  |  |  | X |  |  |
| ILB administration |  |  | X | X | X | X | X |  |  |  |
| Blood sampling PK, HGF, APTT^2^ |  |  | X^3^ |  |  |  | X^3^ |  |  |  |
| Blood sampling for explorative analyses |  | X |  |  | X^4^ |  |  | X |  |  |
| CSF sampling for exploratory analyses |  | X |  |  |  |  |  | X |  |  |
| Adverse events | X^5^ | X^5^ | X^5-6^ | X^6^ | X^6^ | X^6^ | X^6^ | X^6^ | X^6^ | X^6^ |

^1^ For women of child-bearing potential.

^2^ Effect APTT (not safety APTT).

^3^ For detailed time points and time windows on dose administration day, see Table 3.

^4^ For detailed time points and time windows on dose administration day, see Table 4.

^5^ Collection of baseline adverse events starts after the subject signs the ICF and continues until the first administration of ILB at Visit 2.

^6^ Treatment-emergent adverse events (TEAEs) are collected starting from first administration of the IMP (Visit 2) until the last visit (Visit 9).

^7^ IMP dosing/administration should occur within ±3 days from the specified day, with at least 4 days between two IMP administrations.

Table 3 Detailed schedule of events for Visit 2 and Visit 6

| **Visit 2 and Visit 6** |  |  |  |  |  |  |  |  |  |  |
| --- | --- | --- | --- | --- | --- | --- | --- | --- | --- | --- |
| Time points | Pre-dose^1^ | -15 min | 0 | 30 min | 1 h | 2 h | 2.5 h | 3 h | 4 h | 6 h |
| Time windows (min) |  | ± 5 |  | ± 5 | ± 5 | ± 5 | ± 5 | ± 5 | ± 5 | ± 5 |
| Concomitant medication | X |  |  |  |  |  |  |  |  |  |
| Physical examination | X |  |  |  |  |  |  |  |  |  |
| Weight | X^2^ |  |  |  |  |  |  |  |  |  |
| Vital signs | X |  |  |  |  |  |  |  |  |  |
| Forced vital capacity (FVC) | X |  |  |  |  |  |  |  |  |  |
| Haematology, incl PK-INR | X |  |  |  |  |  |  |  |  |  |
| Clinical chemistry | X |  |  |  |  |  |  |  |  |  |
| Pregnancy | X |  |  |  |  |  |  |  |  |  |
| ALSFRS-R and Norris | X |  |  |  |  |  |  |  |  |  |
| Quality of life | X |  |  |  |  |  |  |  |  |  |
| Autonomous symptoms | X |  |  |  |  |  |  |  |  |  |
| ILB administration |  |  | X |  |  |  |  |  |  |  |
| Blood sampling PK, HGF, APTT^3^ |  | X^4^ |  | X | X | X | X | X | X | X |
| Adverse Events / TEAEs | X^5^ | X | | | | | | | | |

^1^ Pre-dose (within 2 hours before administration of IMP).

^2^ Only at Visit 2. For calculation of IMP volume to be administered.

^3^ Time points are based on time from start of IMP administration. APTT for effect of IMP, not safety APTT. For details on blood sampling procedures, see Section 11.4.7.

^4^ Before injection of IMP, additional blood samples for sample dilution and generation of standard curves will be collected, see Section 11.4.6.

^5^ Treatment-emergent adverse events (TEAEs) assessment performed on Visit 6 only. Baseline events are collected on Visit 2.

Table 4 Detailed schedule of events for Visit 4

| **Visit 4** |  |  |  |  |  |  |  |  |  |  |
| --- | --- | --- | --- | --- | --- | --- | --- | --- | --- | --- |
| Time points | Pre-dose^1^ | -15 min | 0 | 30 min | 1 h | 2 h | 2.5 h | 3 h | 4 h | 6 h |
| Time windows (min) |  | ± 5 |  | ± 5 | ± 5 | ± 5 | ± 5 | ± 5 | ± 5 | ± 5 |
| Concomitant medication | X |  |  |  |  |  |  |  |  |  |
| Physical examination | X |  |  |  |  |  |  |  |  |  |
| Vital signs | X |  |  |  |  |  |  |  |  |  |
| Forced vital capacity (FVC) | X |  |  |  |  |  |  |  |  |  |
| Haematology, incl. PK-INR | X |  |  |  |  |  |  |  |  |  |
| Clinical chemistry | X |  |  |  |  |  |  |  |  |  |
| Pregnancy | X |  |  |  |  |  |  |  |  |  |
| ALSFRS-R and Norris | X |  |  |  |  |  |  |  |  |  |
| Quality of life | X |  |  |  |  |  |  |  |  |  |
| Autonomous symptoms | X |  |  |  |  |  |  |  |  |  |
| IMP administration |  |  | X |  |  |  |  |  |  |  |
| Blood sampling (exploratory)^2^ |  | X^3^ |  | X | X | X | X | X | X | X |
| TEAE | X | | | | | | | | | |

^1^ Pre-dose (within 2 hours before administration of IMP).

^2^ Blood samples collected for exploratory objective. Time points are based on time from start of IMP administration.

^3^ Before injection of IMP, additional blood samples for sample dilution and generation of standard curves will be collected, see Section 11.5

# STUDY POPULATION

## Screening log

Potential study subjects will be assessed for inclusion into the study at an initial screening visit. A screening number will be allocated to each patient. Investigators must keep a record of all screened subjects even if they were not subsequently included in the study. This information is necessary to verify that subjects were selected without bias. The reason for screen failure should be stated for all subjects screened but not included. The reason for withdrawal should be stated for all subjects included but not completed.

A patient may be rescreened. If a subject cannot receive the planned dose of IMP within 30 days after screening (*i.e.*, the time interval between signing informed consent until dose administration) the subject should be rescreened before proceeding in the trial. A result from CSF sampling at Visit 1b will be valid for 6 months.

## Number of subjects

15 patients will be included in the study. No formal sample size calculation has been performed for this study. The proposed sample size is considered sufficient in this early phase II development to provide adequate information on the patients.

## Inclusion criteria

To be eligible for inclusion in the study, subjects must fulfil all the following criteria:

1. Willing and able to give written informed consent for participation in the study.
2. Clinical diagnosis of Amyotrophic Lateral Sclerosis (ALS).
3. Male or female patients between 18 to 80 years (inclusive).
4. Forced Vital Capacity (FVC) ≥ 65% of predicted value for gender, height and age at screening.
5. Evaluated with ALSFRS-R and Norris clinical rating scales for at least the past 4 weeks before study drug administration.

## Exclusion criteria

Subjects must not enter the study if any of the following exclusion criteria are fulfilled:

1. Unable to understand information about the study or are expected not to collaborate with the study team.
2. Concurrent serious disease, other than ALS, at the discretion of the Investigator.
3. Pregnancy.
4. Patients of childbearing potential not willing to use adequate double contraception^[[1]](#footnote-2)^ with < 1% failure rate after the screening visit until the last visit.
5. Addiction to drugs or alcohol.
6. Confirmed HIV, hepatitis B or hepatitis C.
7. Known bleeding disorders or abnormal bleeding events.
8. Treatment with anticoagulant drugs warfarin and novel oral anticoagulants (NOAC) within the last 14 days prior to screening.
9. Treatment with Riluzole or Lamotrigine within the last 28 days prior to study drug administration.
10. Hypersensitivity to dextran sulfate.
11. Poor venous access.
12. Patients with clinically significant abnormal PK-INR, fibrinogen, von Willebrand factor and activated partial thromboplastin time (APTT) at screening.

## Prior and concomitant therapy

Patients must not use anticoagulant drugs warfarin and novel oral anticoagulants (NOAC) during the study, and within the last 14 days prior to screening. Patients must not use Riluzole or Lamotrigine during the study, or within the last 28 days prior to study drug administration. Chronic intake of medication including herbal remedies and over the counter drugs on a stable dose for more than two months before screening is allowed. Hormonal contraception is allowed. If there is a need for use of other medication due to intercurrent disease at any time during the study period until end of study the decision will be declared by the patient to the investigator.

## Withdrawal of patients from therapy or assessment

### General withdrawal criteria

Subjects are free to discontinue their participation in the study at any time. Biological samples from the subject will be disposed of, if not already analysed and documented.

Subjects may be discontinued from the study at any time at the discretion of the Investigator for any of the following reasons:

- Severe non-compliance to study protocol procedures
- Significant AEs posing a risk for the subject, including risks of grade B (as stated in Appendix 1).
- Patient contracts a serious disease or has an accident

### Criteria for temporary withdrawal

A subject will be temporarily withdrawn from the study for the following reason:

- Decrease of platelet or neutrophil counts below 50 % of the level at inclusion, until recovery of laboratory findings.

### Criteria for permanent withdrawal

A subject will be permanently withdrawn from the study for the following reasons:

- Withdrawal of informed consent
- in case of pregnancy, planned pregnancy or non-compliance with set requirements for contraception
- if treatment with anticoagulants warfarin/NOAC is necessary
- concomitant treatment with Riluzole or Lamotrigine

### in case of Serious Adverse Events (SAE) that are assessed as possibly or probably related to study treatment, including all risks categorized as of grade C (as stated in Appendix 1).

### Procedures for discontinuation of a subject from the study

A subject who prematurely discontinues participation in the study will always be asked about the reason(s) for discontinuation and the presence of any AEs. If possible, the patient will be seen by the investigator and assessed according to the procedures scheduled for the End-of-study follow-up (Visit 9). Any ongoing AE will if possible be followed as described in ‘Section 12.3.6’

In the event that a patient or their partner becomes pregnant in the period between Visit 2 and Visit 9, the outcome of the pregnancy should if possible be followed.

The reason and date the subject is removed from the study is to be documented in the Case Report Form (CRF) and in their medical records.

9.6.5 Subject replacement

A subject that has not received any IMP treatment will be replaced. Subjects who are prematurely withdrawn from the study for any reason except the occurrence of AEs assessed as possibly or probably related to study treatment may be replaced during the course of the study.

# TREATMENT

## Treatment administration

The investigational medicinal product ILB will be given 1 mg/kg as single short-term s.c. injections in the abdomen, the thigh or the buttock, in that order of priority. The maximum volume to be injected at each injection site is approximately 2 ml and the number of injections per subject may range between 1 and 3 sites depending on the volume to be injected. Each administration should occur within ±3 days from the day specified in Table 2, with at least 4 days between two IMP administrations. After all other visit-specific assessments are completed (Table 2), ILB administration will be performed by the staff at the study site, and the subjects will be observed for at least 3 hours after injection.

## Identity of Investigational Medicinal Product

The IMP contains 20 mg/ml ILB and 9 mg/ml NaCl and is a sterile, colourless to pale yellow solution for subcutaneous injection. The active pharmaceutical ingredient is a type of low molecular weight dextran sulfate (LMW-DS, approx. 20 % sulfate, Mw 5 kDa).

## Packaging and labelling

The IMP (ILB 20 mg/ml) is filled in a 10 ml glass vial sealed with a rubber stopper and a tear-off aluminium cap. The vials will be provided in paper boxes and there will be 10 x 10 ml glass vials in each box. Each vial will be labelled with the information in Figure 2, and each paper box will be labelled with the information in

Figure 3.

Labelling and distribution of ILB to the trial site is done in accordance with local law, trial requirements and Good Clinical Practice.

Figure 2 Information on IMP vial label


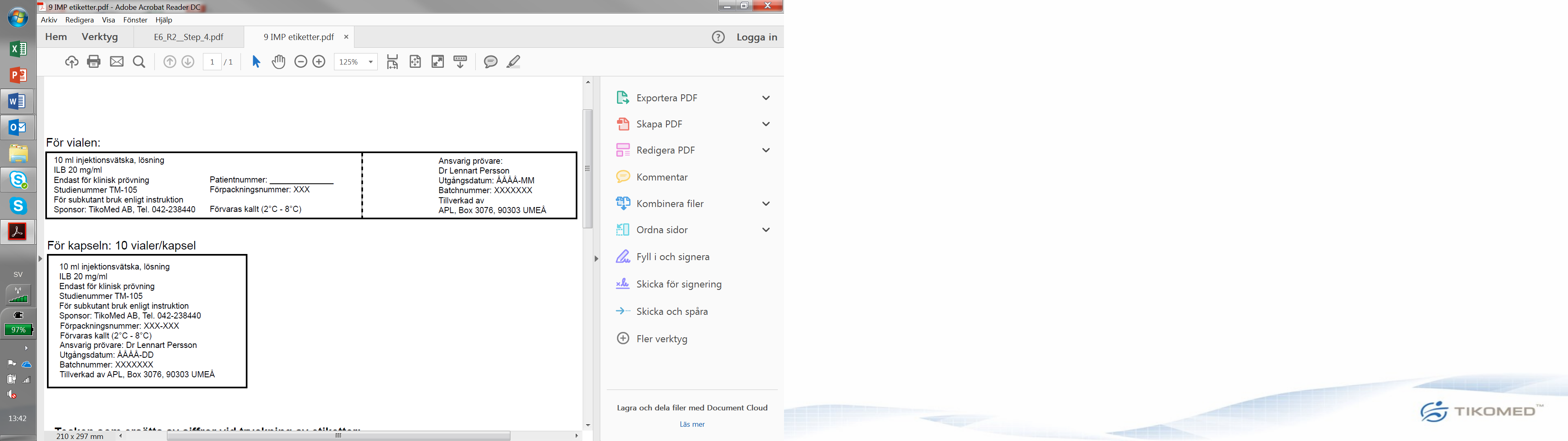


Figure 3 Information on IMP box label


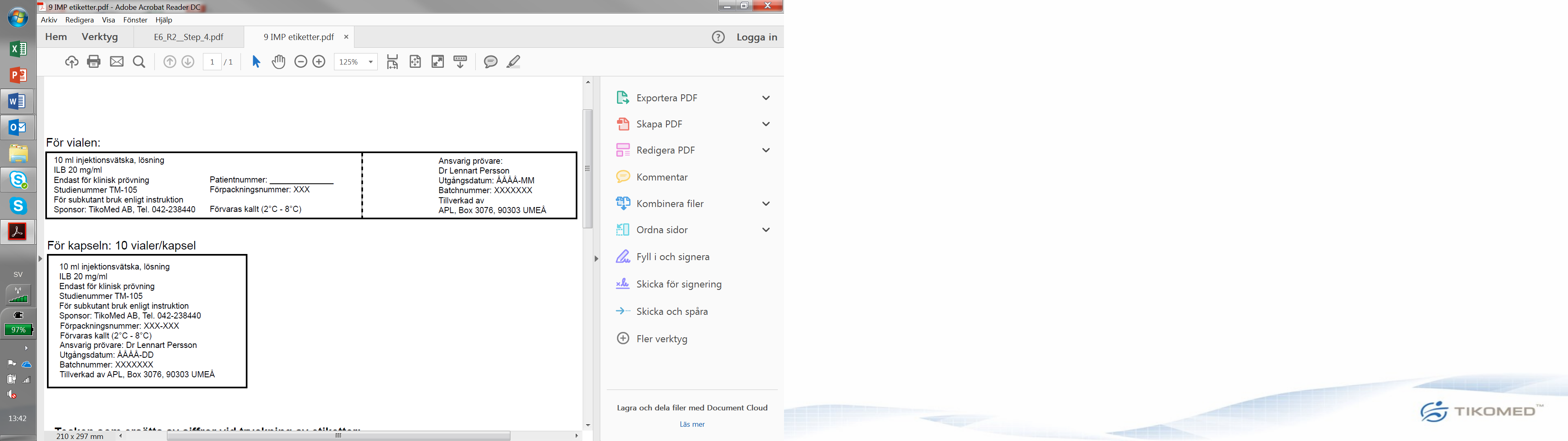


## Conditions for storage

The IMP must be stored at +2°C to +8°C and should not be kept at room temperature for more than one hour prior to dosing.

## Dispensing and accountability

The investigator will maintain a drug dispensing log detailing the dates and quantities of IMP received, dispensed and used by each subject, and study medication returned at the end of the study. Any discrepancies between dispensed and returned IMP must be explained and documented. Unused, partially used and empty ILB vials are to be sent to the Sponsor.

## Treatment compliance

The IMP will be administered at the research clinic under medical supervision to ensure compliance.

# STUDY ASSESSMENTS

The procedures and timing of the study assessments are described in the sections below. For overview, see also the schedule of events in Table 2.

For visits with critical time points for blood sampling, detailed schedule of events are presented in Table 3 (Visit 2 and Visit 6) and Table 4 (Visit 4).

In case of deviations from normal, repeated tests can be performed.

## Recording of data and time windows

The Principal Investigator will provide the Sponsor with all data produced during the study from the scheduled study assessments. He ensures the accuracy, completeness, legibility, and timeliness of the data reported to Sponsor in the CRF and in all required reports.

Pre-dose assessments may be performed within 2 hours prior to dosing. The order of priority for pre-dose sampling and assessments are:

1. Vital signs
2. Safety laboratory tests
3. Efficacy laboratory tests

After ILB administration, it is important that sampling for pharmacokinetics (PK) occurs as close as possible to scheduled time. In order to achieve this, the order of priority is:

1. Blood samples for PK and Hepatocyte Growth Factor (HGF). The time points for PK-HGF sampling will start from the start time of IMP injection.
2. Blood samples for Activated Partial Thromboplastin Time (APTT)

## Demographics and other baseline characteristics

### Informed consent

Information about the aims and procedure for the planned investigation, including information on contraception and medication, will be presented to the patient. Signed informed consent must be obtained before any screening procedures are initiated at Visit 1a.

### Demographic information

The following demographic data will be recorded: gender, age, and ethnic origin.

### Weight and height

Height will be measured at screening (Visit 1a) in patients without shoes and reported in centimetres. The body weight will be recorded at Visit 1, 2 and 7, without shoes in light clothing, and be reported in kilograms. Body Mass Index (BMI) will be calculated from the height and weight recorded at Visit 1 and rounded off to the nearest whole number. The body weight recorded at Visit 2 will be used for calculation of the IMP volume to be administered at Visit 2 to Visit 6.

### Medical history

Relevant medical history will be obtained by interview (Visit 1a).

### Prior and concomitant medication

Relevant prior and concomitant will be obtained by interview (Visit 1a).

### HIV and Hepatitis B/C

Patients will be tested for HIV and hepatitis B and C (serum) at the screening (Visit 1a) to verify that eligibility criteria are met.

### Drug screen

Patients will be tested for drugs of abuse at screening (Visit 1a) to verify that eligibility criteria are met. Urine will be tested for opiates, cocaine, cannabinoids, benzodiazepines and amphetamine. Additional random tests can be performed during the study period.

### Alcohol abuse

Patients will be tested for gamma glutamyltransferas at screening (Visit 1a) to evaluate alcohol abuse.

### Pregnancy

Women of child-bearing potential will be tested for pregnancy (urine) at Visit 1a and Visit 2 to Visit 9.

## Assessments related to the primary endpoint

### Adverse events

AEs (including baseline events) identified using any of the following methods will be recorded:

- AEs spontaneously reported by the subject
- AEs observed by the Investigator or medical personnel
- AEs elicited based on non-leading questions from the Investigator or medical personnel

Adverse events will be handled as described in section 12.

### Physical examination

A complete physical examination will include assessments of the head, eyes, ears, nose, throat (EENT), cardiac, peripheral vascular, pulmonary, musculoskeletal, neurologic, abdominal, lymphatic and dermatologic functions.

### Vital signs

Systolic and diastolic blood pressure (BP), heart rate (pulse) and body temperature will be measured in supine position after 5 minutes of rest using the same method each time.

### Resting 12-lead ECG

Single 12-lead ECGs will be recorded after 10 minutes supine rest using an ECG machine. PQ/PR, QRS, QT and QTcH intervals will be recorded.

### Laboratory safety assessments

Blood samples for analysis of clinical chemistry, haematology and haemostatic assessments parameters will be collected according to local routine and sent to the certified clinical chemistry laboratory at Sahlgrenska University Hospital (SU) and analysed by routine analytical methods.

The following safety laboratory parameters will be assessed:

| **Clinical Chemistry** | **Haematology** |
| --- | --- |
| Sodium | Haemoglobin (Hb) |
| Potassium | Haemoglobin S (only at screening) |
| Chloride | HbA1c (only at screening) |
| Calcium | Red blood cell count (RBC) |
| Albumin | WBC |
| Aspartate aminotransferase (AST) | Differential cell count |
| Alanine aminotransferase (ALT) | Platelets (thrombocytes) |
| Creatine kinase (CK) |  |
| Alkaline phosphatase |  |
| Creatinine | **Haemostatic assessments** |
| Myoglobin | Activated Partial Thromboplastin Time (APTT) |
| C-reactive protein (CRP) | Fibrinogen |
| Total bilirubin  Glucose (non-fasting) | Von Willebrand factor (vW antigen, vW activity + factor VIII) (only at screening) |
|  | Prothrombin Complex-International Normalised Ratio (PK-INR) |

Any remains from the safety laboratory samples will be disposed of after analysis.

## Assessments related to secondary endpoints

### ALSFRS-R and Norris rating scales

Disease severity will be evaluated by the Investigator using the ALSFRS-R and Norris rating scales (Appendix 2-3) in an interview with the patient.

### Biomarkers for neurological diseases, including NFL

Extent of presence of biomarkers for neurological diseases will be evaluated in serum, plasma and CSF sampled on Visit 1b and Visit 7.

The samples, apart from those for Compleasome analysis, will be transferred to Clinical Chemistry at SU before further transfer to Neurochemistry Laboratory Mölndal University Hospital for analysis. Samples for Compleasome analysis will be transferred to Department of Bacteriology at SU. Detailed procedures for sample handling will be provided in a separate lab manual.

The following biomarker parameters will be assessed:

| **In CSF** | **In serum** | **In plasma** |
| --- | --- | --- |
|  | Albumin | Neurofilament Light Chain (NFL) |
| Albumin | IgG | Compleasome |
| IgG | IgM |  |
| IgM |  |  |
| IgG index |  |  |
| IgM index |  |  |
| Tau |  |  |
| Phospho Tau |  |  |
| Neurofilament Light Chain (NFL) |  |  |
| Beta-amyloid |  |  |
| GFAP  Compleasome |  |  |

Any material remaining when the database is closed will be shipped to the Sponsor’s biobank and used for exploratory analyses (Section 11.5), if the patient has consented to this. Otherwise, samples remaining after database lock will be disposed of locally.

### Pulmonary function

The pulmonary function parameter Forced Vital Capacity (FVC) will be assessed with a microspirometer.

### Quality of Life

The patient’s quality of life (QoL) will be evaluated using a Visual Analogue Scale (VAS)-based questionnaire filled out by the patient and, if applicable, a next of kin (Appendix 4-5). The same next of kin must be used throughout the study. If a next of kin does not evaluate the QoL at some or all visits, this will not be regarded as a protocol deviation. Personal information about the next of kin must not be recorded on the QoL questionnaire sheet or in the study database, only in the patient’s medical record.

### Autonomous symptoms

Prevalence and extent of sensory and autonomous symptoms (Appendix 6) will be recorded by the Investigator in an interview with the patient.

### Hepatocyte Growth Factor (HGF) and pharmacokinetic measurement of ILB

Blood samples for HGF and analysis of ILB pharmacokinetics (PK) will be collected through venepuncture or an indwelling venous catheter into a vacutainer tube with citrate at the time points specified in Table 3. The blood tubes will be centrifuged, and the separated plasma is transferred to cryotubes and immediately frozen. Detailed procedures for sample handling will be provided in a separate lab manual.

The time points for HGF and PK blood sampling will start from the start time of injection and be collected within the time windows given in Table 3. Blood samples for sample dilution and generation of standard curves will be collected prior to dose administration at the time point specified in Table 3. The actual time points for blood sampling will be recorded in the CRF and will be used in the data analysis. The pharmacokinetic parameters to be evaluated are listed in Section 16.6.6.

For HGF, one cryotube (A-sample) will be transferred to Clinical Immunology Laboratory at SU for ELISA-based assay of HGF concentration. For PK, one cryotube (A-sample) will be sent to Eurofins Munich to be analysed by a validated method for content of ILB.

Back-up tubes will remain at the trial clinic until the analysis at Clinical Immunology and Eurofins has been successfully completed. If required, back-up tube(s) may also be sent. Any remains from the HGF and PK samples sent to Clinical Immunology and Eurofins, will be disposed of locally after analysis is finished. Any back-up tubes remaining at SU when the database is closed will be shipped to the Sponsor’s biobank and used for exploratory analyses (Section 11.5), if the patient has consented to this. Otherwise, the back-up samples remaining after database lock will be disposed of locally.

### Effect APTT

The IMP has a consistent and dose-dependent effect on Activated Partial Thromboplastin Time (APTT). The samples for analysis of the effect APTT will be collected and processed according to local routine and sent to the certified clinical chemistry laboratory at SU to be analysed by routine analytical methods.

Blood samples for effect APTT will start from the start time of IMP administration and be collected within the time windows given in Table 3. The actual time points will be recorded in the CRF.

## Assessments related to the explorative objectives

A number of possible future exploratory analyses (*e.g.* growth factors, cytokines and biomarkers) may be performed to further elucidate the mechanisms of the IMP and the disease. All future exploratory analyses will be performed after completion of the study and reported separately. The study subjects will be informed about these possible future analyses and give a separate consent to the procedure.

For the exploratory objectives, four extra blood samples (plasma: 2 citrate and 2 EDTA tubes) will be collected at time points specified in Table 2 and Table 4. In addition, 4 ml plasma, 4 ml serum and 4 ml CSF will be collected at Visit 1b and Visit 7.

Plasma and CSF will be prepared according to a separate lab manual and stored at -80°C at the clinic until transportation to the Sponsor’s biobank.

## Total volume of blood and CSF per patient

The total volume of blood taken from each subject during the study is maximum 120 ml per visit and in total maximum 1080 ml during 4 months.

The total volume of CSF taken from each subject during the study is maximum 20 ml per visit (Visit 1b and 7), in total 40 ml.

## Biobank

Unless otherwise noted in Sections 11.2 - 11.5, all samples taken in this study will be kept in Biobank Väst (890) and handled according to the biobank act (Biobankslagen SFS [2002:297](https://lagen.nu/2002:297)) and regulations about biobanks in health service and care. The law regulates the manner in which samples can be stored and used as well as regulation about quality and safety for biobanks. The samples will be stored prior to analysis and coded to protect the identification of the subjects. All samples and the identification list will be kept safely and separated to prevent access of unauthorized persons.

A full chain of custody is maintained for all samples throughout their lifecycle. The sample receiver (the analytical laboratory) keeps full traceability of the samples while in their storage until used, disposed of or transferred to the Sponsor’s biobank, as appropriate. The Sponsor keeps oversight of the entire life cycle through internal procedures, monitoring of study sites and auditing of external laboratory providers.

If a subject withdraws consent to the use of biological samples donated, the samples will be disposed of /destroyed, if not already analysed and documented.

The Principal Investigator will ensure that:

- Subject withdrawal of informed consent is notified immediately to Sponsor.
- Biological samples from the subject, if stored at the research clinic, are immediately identified, disposed of/destroyed and the action is documented.

The Sponsor has to ensure that the laboratory(ies) holding the samples is/are informed about the withdrawn consent immediately and that samples are disposed of/destroyed or returned to the research clinic and the action is documented. All plasma samples transferred to the Sponsor’s biobank will, if not used, be disposed of after 10 years.

# Safety

## Expected effects

An increase in APTT is an expected pharmacological effect which in previous clinical studies with ILB has not corresponded to an increased risk of bleeding. The increase in APTT is dose-dependent and is consistently returned to normal within 6-24 hours depending on the IMP dose administered. Therefore, APTT values above the limit for normal values at the local lab should not be reported as an AE, unless it is still elevated after 24 hours after the last dose administration. However, due to the IMP’s effect on APTT, and results on previous studies in the literature with similar molecules, a conservative approach will be used and patients with bleeding disorders will not be included in the study.

After ILB administration in humans, two cases of mild and transient hypersensitivity reaction have been reported, which were judged to have a causal relationship with the IMP. No actions were taken to treat the events, since the reactions stopped spontaneously. The medical staff at the trial site have extensive experience from early Phase II studies and there are adequate procedures in place to handle adverse reactions such as hypersensitivity reactions.

After subcutaneous administration of ILB 100 mg/ml, local effects have been seen within 10 minutes in terms of mild pain, pruritus, erythema and oedema. The effects were transient and had disappeared within 30 minutes or 4 hours after administration, and were never seen as a problem.

Planned hospitalisations or surgical interventions for a condition that existed before the subject signed the ICF and that did not change in intensity are not SAEs.

See Appendix 1 for a risk management plan.

## Definitions

### Adverse Event (AE)

An AE is any untoward medical occurrence in a patient or clinical investigation subject administered a pharmaceutical product and which does not necessarily have a causal relationship with this treatment. An AE can therefore be any unfavourable and unintended sign (including an abnormal laboratory finding), symptom, or disease temporally associated with the use of a medicinal (investigational) product, whether or not related to the medicinal (investigational) product.

A *baseline event* is any AE in a clinical study subject that occurs after he/she signed the Informed Consent Form (ICF) up until the first administration of IMP.

A *treatment emergent AE* (TEAE) is any AE not present prior to the initiation of IMP administration or any event already present that worsens in either intensity or frequency following exposure to the IMP.

***Causality***

The Investigator is responsible for determining whether there is a causal relationship between an AE and the use of a medicinal product.

All AEs are categorized either as unrelated, possibly related or related, as defined below:

- **Unrelated**: the AE is not reasonable in relation to the use of the medicinal product, or another cause can itself explain the occurrence of the event.
- **Possibly related**: the AE may be explained by the medicinal product and the onset is reasonable in relation to the use of the medicinal product, however there is insufficient information to determine the likelihood of this possibility.
- **Related**: the AE is most likely explained by the medicinal product and the onset is reasonable in relation to the use of the medicinal product.

For a baseline event, a causality assessment is not relevant.

***Severity***

In addition to assessing the relationship of the administration of the investigational product to adverse events, an assessment is required of the intensity (severity) of the event. The following over-all classifications should be used:

- **Mild:** An adverse event which is relatively mild and transient in nature, but can be an annoyance, and does not interfere with normal activities.
- **Moderate:** An adverse event which may be uncomfortable but is not hazardous to health. It may be sufficiently discomforting to interfere with normal activities but does not completely prevent them.
- **Severe:** An adverse event which is incapacitating and/or is a hazard to the subject.

### Adverse Drug Reaction (ADR)

In the pre-approval clinical experience with a new medicinal product or its new usages, particularly as the therapeutic dose(s) may not be established: All noxious and unintended responses to a medicinal product related to any dose should be considered adverse drug reactions. The phrase responses to a medicinal product means that a causal relationship between a medicinal product and an adverse event is at least a reasonable possibility, i.e. the relationship cannot be ruled out.

### Serious Adverse Event (SAE)

Any untoward medical occurrence that at any dose:

- results in death,
- is life-threatening,
- requires inpatient hospitalization or prolongation of existing hospitalization,
- results in persistent or significant disability/incapacity, or
- is a congenital anomaly/birth defect

Medical and scientific judgement should be exercised in deciding whether an event is “serious” and if expected reported is appropriate in other situations, such as important medical events that may not be immediately life-threatening or result in death or hospitalisation, but may jeopardize the subject or may require intervention to prevent one of the other outcomes listed in the definition above. These should also usually be considered SAEs.

If there is any doubt as to whether an AE meets the definition of an SAE, a conservative viewpoint must be taken, and the AE must be reported as an SAE.

### Suspected unexpected serious adverse reaction (SUSAR)

A SUSAR is a serious adverse reaction of which nature or severity is not consistent with the applicable product information (e.g. Investigator's Brochure for an unapproved investigational product or package insert/summary of product characteristics for an approved product).

## Reporting

### Adverse Event (AE)

All AEs occurring during the trial that are observed by the Investigator or reported by the subject, will be recorded by the Investigator or designee on the CRF, whether or not attributed to trial medication.

The following information will be recorded: description, date (and time, if relevant) of start and end, severity, assessment of relatedness to trial medication, action taken and outcome. Follow-up information should be provided as necessary. Any AE with start date on the day of first IMP administration (Visit 2) must be recorded with start time.

### Adverse Drug Reaction (ADR)

AEs considered related to the trial medication as judged by a medically qualified investigator or the Sponsor will be followed either until resolution, or the event is considered stable.

### Serious Adverse Event (SAE)

The investigator must report SAEs to the Sponsor within 24 hours of the Site Study Team becoming aware of the event being defined as serious. An alert that an SAE has occurred should be sent to the Sponsor, by contacting:

| **Sponsor Representative** | **Sponsor’s Medical Expert** |
| --- | --- |
| LINK Safety  E-mail: [safety@linkmedical.se](mailto:safety@linkmedical.se) | Anders Svensson, MD PhD |
|  | Högadalsgatan 2B |
|  | 431 69 GÖTEBORG |
|  | Telephone: 0708 92 92 91 |
|  | Email: anders.svensson@tikomed.com |
|  |  |

An alert must not contain confidential information. The alert should promptly be accompanied by an SAE report using the SAE Reporting Form, sent to the Sponsor by e-mail:

**safety@linkmedical.se**The first report should contain as much information as possible, and if more information about the subject’s condition becomes available, a follow-up report with additional information must be submitted within 24 hours using the same procedure as for the initial report. All SAEs must also be recorded in the patient’s medical record.

Upon receipt, the Sponsor will send a written confirmation of the receipt to the Investigator. The Sponsor will review the SAE in a timely manner, taking into account the reporting time for a potential SUSAR, and assess expectedness using the Reference Safety Information current at the time of event.

If the Sponsor evaluates that the SAE is a SUSAR, this will be reported to the Competent Authority (CA) (EudraVigilance database) and to the Ethics Committee (EC), as outlined below.

### Suspected Unexpected Serious Adverse Reaction (SUSAR)

The Sponsor representative LINK Safety will report all SUSARs to the relevant CA (EudraVigilance database) and to the EC and other parties as applicable. For fatal and life-threatening SUSARs, this will be done no later than 7 calendar days after the Sponsor or delegate is first aware of the reaction. Any additional relevant information will be reported within 8 calendar days of the initial report. All other SUSARs will be reported within 15 calendar days.

### Annual safety report

A safety report will be completed by Sponsor once a year and sent to the relevant CA and the EC. The document will define the time period reported and summarize all occurred serious events (SAEs and SUSARs). The safety report should also always include a summary assessment of the safety of subjects that are still included in the trial and whether the benefit-risk assessment changed since the study was approved.

### Treatment and follow-up of adverse events

Subjects with AEs that occur during the study must be treated according to daily clinical practice at the discretion of the Investigator.

AEs must be followed up until resolution or until the last Follow-up Visit, whichever comes first. At the follow-up visits (Visit 7 to Visit 9), information on new AEs, if any, and stop dates for previously reported AEs must be recorded. AEs on-going at the final follow-up visit (Visit 9) may be followed up until assessed as stable or until resolution as judged by the Investigator.

It is the responsibility of the Investigator to follow up on all SAEs until the subject has recovered, stabilised, or recovered with sequelae, and to report to the Sponsor all relevant new information using the same procedures and timelines as those for the initial report. Relevant information includes discharge summaries, autopsy reports, and medical consultation.

SAEs spontaneously reported by a subject to the Investigator within 30 days after the last follow-up assessment must be handled in the same manner as SAEs occurring during the study. These SAEs will be reported to the Sponsor.

### Pregnancy reporting

Female patients will be instructed to notify the Investigator immediately if they become pregnant during the study. Male patients will be instructed to notify the Investigator immediately if their partner becomes pregnant. Pregnant patients will be withdrawn from further study treatment. The patients will also be instructed to report pregnancies discovered after the last visit if they believe that conception occurred during their participation in the study.

A pregnancy is not an AE unless there is a possibility that the IMP has interfered with the efficiency of any contraceptive measures. However, the Investigator should report pregnancies according to the procedures and timelines described for reporting of SAEs. The pregnancy notification form should be used instead of the SAE form.

Pregnant patients or partners will be followed until the end of the pregnancy. Any complication during the pregnancy will be reported as an AE. The outcome of the pregnancy must be reported on the pregnancy outcome form. Any spontaneous abortion, stillbirth, birth defect/congenital anomaly, death, or other serious infant condition must be reported and followed up as an SAE.

# ETHICAL AND REGULATORY REQUIREMENTS

## Ethical conduct of the study

The study will be performed in accordance with the protocol, with the latest version of the Declaration of Helsinki, with the latest version of Good Clinical Practice (ICH-GCP E6(R2)) and applicable regulatory requirements.

## Ethics and regulatory review

The Principal Investigator is responsible for submission of the final study protocol, including the final version of the Informed Consent Form (ICF) and other information given to subjects e.g. advertisements to the Ethics Committee (EC) for approval. The Principal Investigator is responsible for informing the EC of any amendment to the protocol, in accordance with local requirements. Progress reports and notifications of any serious and unexpected adverse drug reactions will be provided to the EC according to local regulations and guidelines.

The Sponsor is responsible for submission of study documents to the applicable competent authority (CA) according to local regulatory requirements.

Approval must be obtained in writing from both EC and CA before the first subject can be recruited.

## Subject information and consent

It is the responsibility of the Investigator or an authorised associate to give each potential study subject full and adequate verbal and written information about the study. The information will include the objectives and the procedures of the study as well as any risks or inconvenience involved. It will be emphasised that participation in the study is voluntary and that the subject may withdraw from participation at any time and for any reason, without any prejudice. All subjects and, if wanted by the subject, the subject’s next of kin will be given the opportunity to ask questions about the study and will be given sufficient time to consider participation before signing the Informed Consent Form (ICF).

Before performing any study-related procedures the latest version of the ICF must be signed and personally dated by the subject and by the Investigator. A copy of the subject information including the signed ICF will be provided to the subject.

Documentation of the discussion and the date of informed consent must be recorded in the CRF, and in the medical record with, at a minimum, the name of the trial and the date when it was signed. The subject information sheet and the signed ICF should be filed by the Investigator for possible future audits and/­or inspections.

The final approved version of the subject information and ICF must not be changed without approval from the Sponsor and the applicable EC.

## Subject data protection

The ICF will incorporate wording that complies with relevant data protection and privacy legislation. Pursuant to this wording, subjects will authorize the collection, use and disclosure of their study data by the investigator and by those persons who need that information for the purposes of the study.

The ICF will explain that study data will be stored in a computer database, maintaining confidentiality in accordance with national data legislation.

The ICF will also explain that for data verification purposes, authorized representatives of the Sponsor, a regulatory authority or an EC may require direct access to parts of the hospital or practice records relevant to the study, including subjects’ medical history.

The Investigator must file a Subject Identification List which includes sufficient information to link records, *i.e.*, the CRF and clinical records. This list should be preserved for possible future inspections/audits but should not be made available to the Sponsor except for monitoring or auditing purposes.

## Protocol deviations and amendments

Modifications to the signed protocol are only possible through approved protocol amendments and with the agreement of all responsible persons. Details of non-substantial amendments are to be clearly noted in the amended protocol.

A change that concerns; a new trial site, new principal investigator and/or a new informed consent form should only be submitted to the concerned EC.

In case of a substantial protocol amendment (e.g. change of; main purpose of the trial, primary/secondary variable, measurement of primary variable, investigational product, or dosing), the concerned EC and CA must be informed and should be asked for its opinion/approval prior implementation of amended protocol, as to whether a full re-evaluation of the ethical aspects of the study is necessary by the committee. This should be fully documented.

The Investigator must not implement any deviation from, or change to, the protocol without discussion with, and agreement by the Sponsor and prior review and documented approval/favorable opinion of the amendment from the relevant EC and CA, except where it is necessary to eliminate an immediate hazard to study subjects, or where the change(s) involves only logistical or administrative aspects of the study (*e.g.* change in monitor(s), change of telephone numbers).

# Quality control and quality assurance

## Training of study site personnel

It is the responsibility of the Principal Investigator to ensure that all personnel involved in the study are fully informed of all relevant aspects of the study and have a detailed knowledge of and training in the procedures that are to be executed by them. Any new information of relevance to the performance of this study must be forwarded to the staff involved in a timely manner.

The Investigator will keep a list of all personnel involved in the study together with their function and study related duties delegated. A Curriculum Vitae (CV) will be available for all staff delegated study-specific duties.

## Clinical Monitoring

The monitor will have regular contacts with the clinic to verify informed consents of participating subjects, to confirm that facilities remain acceptable, that the investigational team is adhering to the protocol and GCP, that data are being accurately recorded in the CRFs, to verify inclusion/exclusion criteria, study main endpoints, check AE/SAE reporting and that therapy accountability is being carried out. The investigator should ensure that all persons assisting with the trial are adequately informed and trained about the protocol, the investigational product and their trial related duties and functions. The monitor will check that training has been performed and that this is documented. The monitor will also ensure source data verification (comparison of the data in the CRF with the medical records and other source data). The monitor must have direct access to source data. The extent of monitoring will be defined in a monitoring plan.

## Audits and inspections

The purpose of an audit or inspection is to systematically and independently examine all study-related activities and documents, to determine whether these activities were conducted, and data were recorded, analysed, and accurately reported according to the protocol, current ICH-GCP guidelines and any applicable regulatory requirements. The Investigator will permit trial-related audits or inspections at the research clinic, including Source Data Verification (SDV), by authorised representatives of Sponsor, a CA, or an EC. The Investigator must ensure that all source documents are accessible for auditing and inspection. The Investigator is required to inform the Sponsor immediately of an inspection requested by a CA or an EC.

# DATA MANAGEMENT

All data should be recorded, handled and stored in a way that allows its accurate reporting, interpretation and verification.

## Source data

Source data are original documents, data and records. The investigator must, for each subject in the study, maintain adequate and accurate source documents, such as medical records, ICF, completed rating scale forms, etc. Changes to source data should be traceable, should not obscure the original entry, and should be explained if necessary. A source data verification log will be included in the Investigator Study File (ISF). The investigator must ensure that all source documents are accessible for monitoring.

## Case Report Form

The investigator will ensure that all data collected in the study are recorded in the CRF in a timely manner according to any instructions provided.

An electronic Case Report Form (CRF) will be used for data collection. Only the Investigator or persons authorised by the Investigator are allowed to make entries to the CRF. The Investigator will ensure that the data is recorded in a timely manner and in accordance with the instructions provided. The Investigator is responsible for the accuracy and completeness of the data entered and sign off the completed CRF. A copy of the completed CRF will be archived at the study site.

## Data Management Plan

Data management refers to the activities for efficient and correct entering of subject information into a database and confirming data accuracy. Detailed information on data management will be described in a study-specific Data Management Plan (DMP) and Data Validation Plan (DVP).

Staff designated by Gothia Forum and working on behalf of the Sponsor will review the data entered into the CRFs by investigational staff for completeness and accuracy and instruct the site personnel to make any required corrections or additions. Queries are issued electronically. Designated investigator site staff is required to respond to the query and confirm or correct the data.

At the conclusion of the study, the occurrence of any protocol deviations will be determined. After these actions have been completed and the database has been declared to be complete and accurate, it will be locked and available for data analysis.

## External Data

External data consists of data that is not recorded in CRFs. Data may be received in electronic format or paper printout. Key variables are defined in order to uniquely identify each sample record. File and data formats are agreed with the external data provider. Any electronically transferred data must contain origin, date created, date sent and number of records at minimum. External data will be entered into the CRF by the Investigator or designee.

## Archiving

The Principal Investigator is responsible for maintaining essential documents, (as defined in ICH E6 GCP, Section 8) for at least 10 years after finalisation of the Clinical Study Report (CSR), in accordance with Swedish regulation/law (Chapter 10, 3 § in LVFS 2011:19). This includes any original source documents related to the study, the Subject Identification List (providing the sole link between named subject source records and anonymous CRF data), the original signed ICFs and detailed records of disposition of IMP.

It is the responsibility of the Sponsor to inform the Investigator/institution as to when these documents no longer need to be retained.

The Sponsor will archive the *Trial Master File* in accordance with ICH E6 GCP, Section 8 and applicable regulatory requirements.

# STATISTICAL METHODS AND DETERMINATION OF SAMPLE SIZE

The principal features of the statistical analysis to be performed are described in this section. A more technical and detailed elaboration of the principal features will be prepared before study data analysis in a separate Statistical Analysis Plan (SAP) which will be signed and approved by the sponsor.

## General

Pharmacokinetic data will be presented using summary statistics. Data will be presented in terms of number (N), arithmetic mean, standard deviation (SD), median, minimum and maximum value. In addition, for the parameters AUC and Cmax the geometric mean and coefficient of variation (CV) will be presented.

Categorical data will be presented as counts and percentages. When applicable, summary data will be presented by assessment time. Individual subject data will be listed by subject number and, where applicable, by assessment time.

## Sample size determination

No formal sample size calculation has been performed for this study. The proposed sample size is considered sufficient in this early phase II development to provide adequate information on the patients.

## Analysis data sets

The full analysis set for efficacy analysis is defined as all subjects who took at least one dose of IMP and with at least one efficacy measurement.

The safety analysis set will consist of all subjects who took any IMP.

No Per Protocol Set will be created and analysed.

## Description of study population

### Demographics and other baseline characteristics

Demographics and other baseline characteristics will be presented using summary statistics.

### Medical history and prior/concomitant medication

Medical/surgical history and prior/concomitant medications will be presented by descriptive statistics and listings.

### Study termination

The reason for study termination and number of subjects who completed the study will be presented with frequency tables.

## Analysis of primary endpoints

### Vital signs (blood pressure, heart rate and body temperature).

Vital signs (blood pressure, heart rate and body temperature) will be presented by visit for each parameter and subject and summarised using summary statistics including absolute and percent change since baseline.

### Physical examination

Abnormal findings will be specified and presented by subject and summarised by frequency tables.

### ECG

All ECG data will be listed for each subject and summarised as the vital signs parameters including absolute and percent change since baseline using summary statistics. In addition, ECGs will be categorised as: “normal, abnormal, not clinically significant”, or “abnormal, clinically significant” (as judged by the Investigator) and summarised by visit.

### Safety laboratory analyses

Safety laboratory data will be presented by visit for each parameter and subject and summarised using summary statistics including absolute and percent change since baseline.

#### AEs

AEs and SAEs will be recorded from start of IMP administration until the end of study follow-up visit (Visit 9). AEs that occur before first treatment with IMP will be reported separately as baseline events.

All AEs will be described in terms of treatment/dose at which they occurred. AEs will be summarised by Preferred Term (PT) and System Organ Class (SOC) using the MedDRA vocabulary. The number of subjects reporting AEs, and the number of AEs reported will be presented. The events will be tabulated by SOC, PT and by severity and relationship to IMP. SAEs will also be presented in separate tabulations. The number of subjects experiencing an AE in connection to the treatment will be presented using descriptive statistics.

## Efficacy endpoints

### Functional rating with ALSFRS-R and Norris scales

Both these scales will be reported according to the manuals with changes from baseline using summary statistics.

### Evaluation of biomarkers for neurological disorders

All biomarkers will be presented using summary statistics with changes from baseline for each visit.

### Pulmonary function (FVC)

The FVC values at baseline and subsequent visits will be presented using summary statistics with changes from baseline.

### Levels of Hepatocyte Growth Factor (HGF)

The levels of HGF will be presented by subject and assessment time using summary statistics with changes from baseline.

### Changes in APTT (effect APTT)

The APTT will be presented by subject and assessment time using summary statistics with changes from baseline.

### Pharmacokinetics of ILB

Individual plasma concentration-time data of ILB will be evaluated using a validated Phoenix WinNonlin software. The pharmacokinetic (PK) analysis (non-compartmental analysis, NCA) will consist of assessment of PK parameters including at least: C_max_, t_max_, t_last_, AUC_last_, AUC_tau_, AUC_(0-t)_, AUC_inf_, t_½_, λz, Rac_obs_, if data permits. Other additional parameters may be assessed as considered appropriate.

## Statistical/analytical issues

### Adjustments for covariates

Not applicable.

### Handling of dropouts or missing data

There will be no analysis done to correct for missing data such as Last Observation Carried Forward, *i.e.* only data present will be used in the analyses.

### Multiple comparison/multiplicity

Not applicable

### Interim analyses and data monitoring

An interim analysis may be performed after half of the study population (6-8 patients) have completed visit 7 to assess futility and safety on the group level. Analysis will be performed for the safety variables, disease severity, biomarkers for neurological diseases including NFL and pulmonary function.

# insurance and financing

## Insurance

The Sponsor agrees to indemnify and hold the Investigator free of harm from any claim, whether based on legal principles or on generally accepted liability standards within the pharmaceutical industry, made against him/her by reason of personal injury, including death, to any person arising out of or connected with the performance of the study to the extent the injury is not cause by:

- Failure by the Investigator to adhere to the terms of the study protocol
- Failure by the Investigator to comply with any applicable governmental regulations
- Malpractice, negligence or wilful malfeasance by the Investigator

The sponsor’s responsibility is covered by the insurance “LIF Läkemedelsförsäkring”.

The study subjects are covered by the Swedish Patient Injury Act and the Pharmaceutical Insurance (<http://lff.se/>). The insurance also covers the sponsor’s liability under law and generally accepted liability standards within the pharmaceutical industry towards any third parties, including subjects as sponsor of the study. The study will not be initiated before proof of this insurance is fulfilled.

In addition, all subjects are covered by the general insurance for patients treated at Swedish medical instances (“Patientförsäkringen”).

## Financing

Details regarding economy will be separately addressed and agreed between the Sponsor and the clinical hospital research unit, the investigators and consultants for medical and clinical management before initiation of the study.

# Study timetable and discontinuation

The study will commence as soon as the CA and the EC have approved the study, as soon as patients have been recruited and as soon as contracts between the Sponsor and all parties have been signed.

The end of the clinical part of the study is defined as the last visit of the last subject participating in the study.

The study is expected to start in Quarter 2, 2018 and to be completed by Quarter 4, 2019.

The Sponsor reserves the right to discontinue the study at any time, but intends only to exercise this right for valid scientific or administrative reasons.

After such a decision, the Investigator must inform all participating subjects and perform relevant assessments, preferably according to the scheme for the final assessments. All delivered and unused study products and other study materials must be returned and all CRFs completed as far as possible.

In the case of early termination, the sponsor must notify the end of the trial to the CA and the EC immediately and at the latest within 15 days after the trial is halted, clearly explain the reasons, and describe follow-up measures, if any, taken for safety reasons.

# Publication policy

As soon as the clinical study is approved by the CA the study is automatically registered in a publicly accessible database before recruitment of the first subject.

After completion of the study, the sponsor will prepare a Clinical Study Report (CSR) in cooperation with the Investigator. The sponsor is responsible for submitting the final version of the CSR to the CA within 12 months after the end of trial notification.

The CSR will form the basis for manuscripts intended for publication in a medical journal. Attempts to publish negative or inconclusive as well as positive results must be made, or otherwise made publicly available. Formal presentation or publication of data collected in this study should be considered as a joint publication by the sponsor and the clinical investigators. The Principal Investigator will be asked to be a part of the authorship. Authorship will be determined by mutual agreement, where one part will be lead author and the others; co-authors. Before any publication (oral or written) of the results, the co-authors will be given 30 days for review and comment on the manuscript. If the lead author has not submitted the results for publication within 6 months after completion of the final CSR, the co-authors have the right to publish as lead authors. In this event, the other part is considered co-author and will be given 30 days to review and comment on the manuscript before it is submitted to a journal. If the findings of the study are of a varying character with diverse matters of results, the data should be made public in several publications with different aspects of the results. In that case, the order of the authors of the different publications will be made in relation to the contribution of work made by the different authors according to the international rules of publication. The costs of publication will be covered by the Sponsor.

# APPENDIX

## Appendix 1. Risk management

| **Risk** | **Grade** | **Clinical sign/value** | **Treatment plan** |
| --- | --- | --- | --- |
| Lung function  Microspirom. | A | No particular signs. | None |
|  | B | Results outside normal ref. range | Observation |
|  | C | Variation >10% between time points | Observation/additional measurements and follow-up |
| Platelet depletion | A | No particular signs. | None |
|  | B | A reduction of >50% from baseline platelet count or ˂ 150x10^9^/L. | Observation |
|  | C | ˂50x10^9^/L, petechial or mucosal bleedings | Observation, consider optimal intervention |
| Bleeding | A | No particular signs. | None |
|  | B | A reduction of haemoglobin of >20g/L, but without clinical signs such as a rise in pulse or drop in blood pressure.  Surgicutt > 900s. | Observation |
|  | C | A reduction of haemoglobin of >20g/L together with clinical signs such as a rise in pulse or drop in blood pressure. Apparent bleeding. | Observation.  1. Stop further administration of ILB.  2. Buffer and/or plasma expander infusion.  3. Transfusion of erythrocytes, fresh frozen plasma and platelets.  4. Administration of Prothrombin complex or FVIIa (NovoSeven®).  5. Surgical intervention if severe bleeding cannot be stopped by steps 1-4 |
| Allergic reactions | A | No particular signs. | None. |
|  | B | Clinical symptoms in combination with blood pressure drop (systolic ˂100) or pulse below 50 beats/min.  Irritation of airways. | Observation. Optimal treatment with adrenaline or steroids. |
|  | C | Blood pressure drop (systolic ˂80) or pulse below 45 beats/min.  Affected breathing. | Try to differentiate from bleeding. Consider optimal treatment with adrenaline and steroids. |
| Out of reference values / Adverse Event (AE) | A | Safety parameters outside the reference ranges/other clinical findings which constitute a medical significant risk. | Observation/treatment suitable for treating the AE condition. |
|  | B | Reaction defined as a SAE. | The event will be observed and reported according to protocol. PI is responsible for the evaluation. |

## Appendix 2: ALSFRS-R Rating Scale

Svensk version av ALSFRS-R Poäng 4-3-2-1-0; 4 bäst, 0 sämst funktion

1. Tal

Normal talprocess

Möjligen talproblem

Begripligt vid upprepning

Tal kombinerat med icke röstkommunikation

Förlust av användbart tal

1. Salivation

Normal

Lindrig men definitivt överdrivet med saliv i munnen, kan ha nattlig dräggling

Måttlig överdriven saliv, kan ha minimal dräggling

Utmärkande överdriven salivation med en del dräggling

Utmärkande dräggling; behov av konstant pappers- eller tygnäsduk

1. Sväljning

Normal matvana

Tidig ätproblematik- tillfälligt svälja fel och storkna

Konsistensförändring av maten

Behov av tillägg med sondmatning

Fullständig parenteral eller enteral matning

1. Handskrift

Normal

Sakta eller slarvig; alla ord är läsliga

Alla ord är inte läsliga

Kan greppa en penna men kan inte skriva

Kan inte greppa en penna

- 1. Skära mat och använda matredskap (patienter utan stomi)

Normal

Lite långsam och klumpig, men behöver inte hjälp

Kan skära det mesta av maten, men klumpigt och långsamt; lite hjälpbehov

Maten måste skäras av någon, men kan äta långsamt

Behöver matas

- 1. Skära mat och använda matredskap (patienter som har stomi)

Normal

Klumpig men kan genomföra alla manipulationer självständigt

En del hjälpbehov med tillslutning och med fästanordning

Ger minimal assistens till vårdgivare

Oförmögen att utföra någon del av arbetsuppgiften

1. På/avklädnad och hygien

Normal funktion

Oberoende och fullständig självhjälp med kraftansträngning eller avtagande effektivitet/skicklighet

Återkommande assistens eller ersättande metoder

Behov av närvarande vid självhjälp

Totalt beroende

1. Vändning i sängen och justering av sängkläder

Normal

Något långsam och klumpig, men inte behov av hjälp

Kan vända sig själv eller justera lakanet, men med stora svårigheter

Kan initiera men inte vända sig eller justera lakan själv

Hjälplös

1. Gång

Normal

Lätta förflyttningssvårigheter

Gå med assistens

Ingen egen förflyttningsfunktion

Ingen meningsfull benfunktion

1. Gå i trappa

Normal

Långsam

Lätt ostadighet eller trötthet

Behov av assistens

Kan inte göra det

1. Dyspné

Ingen

Förekommer vid gång

Förekommer vid en eller flera av följande: äta, bada, på/avklädnad (ADL)

Förekommer vid vila, svårighet med andning vid sittande eller liggande

Betydelsefull svårighet, med övervägning om användning av mekaniskt respiratoriskt stöd

1. Ortopné

Ingen

En del svårigheter under sömn på natten med otillräckliga andetag. Använder inte rutinmässigt mer än två kuddar

Behöver extrakuddar för att kunna sova

Kan endast sova sittande

Kan inte sova utan mekanisk ventilation

1. Respiratorisk insufficiens

Ingen

Intermittent användning av BiPAP

Kontinuerlig användning av BiPAP under natten

Kontinuerlig användning av BiPAP under dagen och natten

Invasiv mekanisk ventilation genom intubation eller tracheostomi

Hur många år är det sedan första symtom? År:________ Totalpoäng:__________

## Appendix 3: Norris Rating Scale

**ALS**

1. Hålla upp huvudet 3=Normal, 2=Nedsatt, 2=svag, 0=saknas
2. Tugga mat
3. Svälja
4. Tala
5. Vända sig i sängen
6. Sitta upp
7. Tömma tarmen/blåsan
8. Andas
9. Hosta
10. Skriva sitt namn
11. Knäppa knappar/blixtlås
12. Äta själv
13. Resa sig upp själv
14. Lyfta bok eller bricka
15. Lyfta gaffel, penna
16. Ändra läge av arm
17. Gå i trappor, en våning
18. Gå ett kvarter
19. Gå över rummet
20. Gå med assistans
21. Stå upp
22. Ändra läge av ben
23. Armreflexer Normal=3, Hyper/Hypo=2 Saknas=1, Klonus=0
24. Benreflexer
25. Masseterreflex Saknas=3, Finns=2 Hyper=1, Klonus=0
26. Babinski höger Flexion=3 Ingen rörelse=2 Odeciderad = 1, Extension= 0
27. Babinski vänster
28. Fascikulationer Inga =3, Lätta= 2 Måttligt = 1, Uttalade = 0
29. Atrofier: ansikte, tunga
30. Atrofier: armar, skuldror
31. Atrofier, ben, höfter
32. Emotionellt labil
33. Uttröttbarhet Ingen eller lätt = 2 Måttlig eller uttalad = 0
34. Rigiditet i benen

Summa________________ (Max 100)

## Appendix 4: Livskvalitet - försöksperson

**Livskvalitet - Försöksperson/Närstående: Egentestning**

Skattning av välbefinnande

Uppskatta **ditt** välbefinnande genom att sätta ett lodrätt streck på linjen där du tycker att det passar bäst in på dig just nu. Linjen sträcker sig från mycket dåligt till mycket bra.

Datum_____________ Försöksperson/Närstående______________

Hur skattar du ditt allmänna välbefinnande?

Mycket dåligt ­­­_____­_____________________________________________ Mycket bra

mm:__ __ __

Hur skattar du ditt fysiska välbefinnande?

Mycket dåligt ­­­_____­_____________________________________________ Mycket bra

mm:__ __ __

Hur skattar du ditt psykiska välbefinnande?

Mycket dåligt ­­­_____­_____________________________________________ Mycket bra

mm:__ __ __

## Appendix 5: Livskvalitet - närstående

**Livskvalitet - Närstående/Närstående**

Skattning av välbefinnande

Uppskatta **din närståendes** välbefinnande genom att sätta ett kryss på linjen där du tycker att det passar bäst in på honom/henne just nu. Linjen sträcker sig från mycket dåligt till mycket bra.

Datum______________ Försöksperson / Närstående_________________

Hur skattar du din närståendes allmänna välbefinnande?

Mycket dåligt ­­­_____­_____________________________________________ Mycket bra

mm:__ __ __

Hur skattar du din närståendes fysiska välbefinnande?

Mycket dåligt ­­­_____­_____________________________________________ Mycket bra

mm:__ __ __

Hur skattar du din närståendes psykiska välbefinnande?

Mycket dåligt ­­­_____­_____________________________________________ Mycket bra

mm:__ __ __

## Appendix 6: Autonoma och sensoriska symtom

0= ej; 1=Något; 2 = Måttligt; 3=Mycket

Autonoma och sensoriska symtom

| **Visit** | **1a** | **2** | **3** | **4** | **5** | **6** | **7** | **8** | **9** |
| --- | --- | --- | --- | --- | --- | --- | --- | --- | --- |
|  | Screening | Dosing | Dosing | Dosing | Dosing | Dosing | Follow-up | Follow-up | End of study follow-up |
| **Day** | -30 to -7 | 1 | 8 | 15 | 22 | 29 | 36 | 50 | 99 |
| **Time window** | ±3 | ±3 | ±3 | ±3 | ±3 | ±3 | ±3 | ±7 | ±7 |
| Smärta |  |  |  |  |  |  |  |  |  |
| Parestesier |  |  |  |  |  |  |  |  |  |
| Frusenhet |  |  |  |  |  |  |  |  |  |
| Svettningar |  |  |  |  |  |  |  |  |  |
| Obstipation |  |  |  |  |  |  |  |  |  |
| Frekvent avföring |  |  |  |  |  |  |  |  |  |
| Miktionssvårigheter |  |  |  |  |  |  |  |  |  |
| Frekventa miktioner |  |  |  |  |  |  |  |  |  |
| Domningar |  |  |  |  |  |  |  |  |  |
| Yrsel |  |  |  |  |  |  |  |  |  |
| Trötthet |  |  |  |  |  |  |  |  |  |
| Uttröttbarhet |  |  |  |  |  |  |  |  |  |
| Insomningsproblem |  |  |  |  |  |  |  |  |  |
| Frekventa uppvaknanden |  |  |  |  |  |  |  |  |  |
| Hjärtklappning |  |  |  |  |  |  |  |  |  |
| Oregelbunden hjärtrytm |  |  |  |  |  |  |  |  |  |

# Study protocol agreement

I have read the preceding protocol entitled:

*A single-centre, open single-arm study where the safety, tolerability and efficacy of subcutaneously administered ILB will be evaluated in patients with Amyotrophic Lateral Sclerosis.*

I agree to conduct the study according to this protocol and to comply with its requirements, subject to ethical and safety considerations.

I understand that, should the sponsor decide to prematurely terminate or suspend the study for whatever cause, such decision will be communicated to me in writing. Conversely, should I decide to withdraw from execution of the study, I will immediately communicate such a decision to the sponsor.

Both parties agree not to publish all or any part of the results of the study carried out under this protocol, without the prior written consent of the other party.

Protocol Version: 1.5, June 25, 2019

Principal Investigator Name: Lennart Persson

Signature: Date:

Sponsor’s representative: Lars Bruce

Signature: Date

# References

1. Mitchell, J.D. and G.D. Borasio, *Amyotrophic lateral sclerosis.* Lancet, 2007. **369**(9578): p. 2031-41.

2. Persson, L.I., H. Ryberg, and L.E. Rosengren, *Correlation between CSF levels of neuronal- and glial-specific proteins versus clinical course and prognosis in amyotrophic lateral sclerosis.* Amyotroph Lateral Scler, 2003. **4**(12).

3. Rosengren, L.E., et al., *Patients with amyotrophic lateral sclerosis and other neurodegenerative diseases have increased levels of neurofilament protein in CSF.* J Neurochem, 1996. **67**(5): p. 2013-8.

4. Ryberg, H., H. Askmark, and L.I. Persson, *A double-blind randomized clinical trial in amyotrophic lateral sclerosis using lamotrigine: effects on CSF glutamate, aspartate, branched-chain amino acid levels and clinical parameters.* Acta Neurol Scand, 2003. **108**(1): p. 1-8.

5. Gordon, P.H. and H. Mitsumoto, *Chapter 20 Symptomatic therapy and palliative aspects of clinical care.* Handb Clin Neurol, 2007. **82**: p. 389-424.

6. Olsson, A.G., et al., *Well-being in patients with amyotrophic lateral sclerosis and their next of kin over time.* Acta Neurol Scand, 2010. **121**(4): p. 244-50.

7. Olsson, A.G., et al., *Differences in quality of life modalities give rise to needs of individual support in patients with ALS and their next of kin.* Palliat Support Care, 2010. **8**(1): p. 75-82.

8. Ozanne, A.G. and L.I. Persson, *Correlations in health status between estimates of families of people with amyotrophic lateral sclerosis and estimates of staff.* Palliat Support Care, 2013. **11**(3): p. 183-9.

9. Lee, J.R., J.F. Annegers, and S.H. Appel, *Prognosis of amyotrophic lateral sclerosis and the effect of referral selection.* J Neurol Sci, 1995. **132**(2): p. 207-15.

10. Traynor, B.J., et al., *Effect of a multidisciplinary amyotrophic lateral sclerosis (ALS) clinic on ALS survival: a population based study, 1996-2000.* J Neurol Neurosurg Psychiatry, 2003. **74**(9): p. 1258-61.

11. Miller, R.G., et al., *Riluzole for amyotrophic lateral sclerosis (ALS)/motor neuron disease (MND).* Cochrane Database Syst Rev, 2007(1): p. CD001447.

12. Lacomblez, L., et al., *Dose-ranging study of riluzole in amyotrophic lateral sclerosis. Amyotrophic Lateral Sclerosis/Riluzole Study Group II.* Lancet, 1996. **347**(9013): p. 1425-31.

13. Bensimon, G., L. Lacomblez, and V. Meininger, *A controlled trial of riluzole in amyotrophic lateral sclerosis. ALS/Riluzole Study Group.* N Engl J Med, 1994. **330**(9): p. 585-91.

14. Walley, T., *Neuropsychotherapeutics in the UK: what has been the impact of NICE on prescribing?* CNS Drugs, 2004. **18**(1): p. 1-12.

15. Gordon, P.H., *Amyotrophic Lateral Sclerosis: An update for 2013 Clinical Features, Pathophysiology, Management and Therapeutic Trials.* Aging Dis, 2013. **4**(5): p. 295-310.

16. Maina, F. and R. Klein, *Hepatocyte growth factor, a versatile signal for developing neurons.* Nat Neurosci, 1999. **2**(3): p. 213-7.

17. Tonges, L., et al., *Hepatocyte growth factor protects retinal ganglion cells by increasing neuronal survival and axonal regeneration in vitro and in vivo.* J Neurochem, 2011. **117**(5): p. 892-903.

18. Ishigaki, A., et al., *Intrathecal delivery of hepatocyte growth factor from amyotrophic lateral sclerosis onset suppresses disease progression in rat amyotrophic lateral sclerosis model.* J Neuropathol Exp Neurol, 2007. **66**(11): p. 1037-44.

19. Warita, H., et al., *[Clinical translation of hepatocyte growth factor for amyotrophic lateral sclerosis].* Rinsho Shinkeigaku, 2012. **52**(11): p. 1214-7.

20. Sun, W., H. Funakoshi, and T. Nakamura, *Overexpression of HGF retards disease progression and prolongs life span in a transgenic mouse model of ALS.* J Neurosci, 2002. **22**(15): p. 6537-48.

21. Pan, W., et al., *Permeation of hepatocyte growth factor across the blood-brain barrier.* Exp Neurol, 2006. **201**(1): p. 99-104.

22. Tsuboi, Y., et al., *Hepatocyte growth factor in cerebrospinal fluid in neurologic disease.* Acta Neurol Scand, 2002. **106**(2): p. 99-103.

23. Schmidt, P., et al., *Low molecular weight dextran sulfate is well tolerated in humans and increases endogenous expression of islet protective hepatocyte growth factor.* Transplantation, 2008. **86**(11): p. 1523-30.

24. Henriques, A., C. Pitzer, and A. Schneider, *Neurotrophic growth factors for the treatment of amyotrophic lateral sclerosis: where do we stand?* Front Neurosci, 2010. **4**: p. 32.

25. Barritault, D., et al., *RGTA^®^ or ReGeneraTing Agents mimic heparan sulfate in regenerative medicine: from concept to curing patients.* Glycoconjugate Journal, 2017. **34**(3): p. 325–338.

26. Tadic, V., et al., *Sigma 1 receptor activation modifies intracellular calcium exchange in the G93A(hSOD1) ALS model.* Neuroscience, 2017. **359**: p. 105-118.

27. Dupuis, L., et al., *Energy metabolism in amyotrophic lateral sclerosis.* Lancet Neurol, 2011. **10**(1): p. 75-82.

28. Guo, Z., et al., *ALS-linked Cu/Zn-SOD mutation impairs cerebral synaptic glucose and glutamate transport and exacerbates ischemic brain injury.* J Cereb Blood Flow Metab, 2000. **20**(3): p. 463-8.

29. Waldemar, G., et al., *Focal reductions of cerebral blood flow in amyotrophic lateral sclerosis: a [99mTc]-d,l-HMPAO SPECT study.* J Neurol Sci, 1992. **107**(1): p. 19-28.

30. Zhong, Z., et al., *ALS-causing SOD1 mutants generate vascular changes prior to motor neuron degeneration.* Nat Neurosci, 2008. **11**(4): p. 420-2.

31. Miyazaki, K., et al., *Early and progressive impairment of spinal blood flow-glucose metabolism coupling in motor neuron degeneration of ALS model mice.* J Cereb Blood Flow Metab, 2012. **32**(3): p. 456-67.

1. Combined (estrogen and progestogen containing) hormonal contraception associated with inhibition of ovulation (oral, intravaginal, transdermal), progestogen-only hormonal contraception associated with inhibition of ovulation (oral, injectable, implantable), intrauterine device (IUD), intrauterine hormone-releasing system (IUS), bilateral tubal occlusion, vasectomised partner, abstinence from heterosexual intercourse. [↑](#footnote-ref-2)
