# Supplementary material for: A phase II open label clinical study of the safety, tolerability and efficacy of ILB® for Amyotrophic Lateral Sclerosis
Source: PLoS One. 2022 May 25;17(5):e0267183. doi: 10.1371/journal.pone.0267183 (PMC9132272; doi:10.1371/journal.pone.0267183)
Supplement: S2 Appendix — (DOCX) [file pone.0267183.s002.docx]

Ann Logan et al. A phase II open label clinical study of the safety, tolerability and efficacy of ILB^®^ for Amyotrophic Lateral Sclerosis

**Supplementary Methods**

The trial is a phase II exploratory trial. There was no formal sample size estimate or power calculation. The sample size was determined empirically and reflects the exploratory nature of the trial and the rarity of ALS (which is an orphan disease; SPIRIT 2013 statement^1^). However, at the insistence of the reviewers and editor we have explored whether the trial design would have been admissible using validated approaches to phase II clinical trial design^2,3^.

For design assumptions in this exploration we did NOT use the actual outcome of the trial. Instead, we relied on what was known in the literature about ALS trial outcomes at the time (high placebo effect and acceptable SAE rate for riluzole). The efficacy expectations would have been a reasonable, albeit ambitious, for a new drug.

As indicated in the main methods section, the trial was planned as a single arm phase II trial, where response rate is an acceptable regulatory end point^4^. However, the definition of treatment goals (definition of positive response) in neuropsychiatric conditions is more complex than in cancer. Expectations depend on the disease, its progression, heterogeneity, definitions of clinically meaningful measures of effect and the likelihood of a placebo effect^5^. As there is no known disease modifying treatment for ALS, there are no normative data that could guide drug response expectations. Additionally, the definition of therapeutic goals will have a significant impact; if phase II expectations are too low, there is a significant risk that a phase III trial may not show a significant treatment effect and *vice versa*, if expectations in phase II are too high, a drug that may be worthy of further development may fail before reaching phase III^6^.

The ALSFRS is an accepted measure of functional deficits seen in ALS patients. The disease is progressive and the decline in the ALSFRS is relentless. There is no spontaneous long-term improvement on ALSFRS in ALS patients. Therefore, no decline in this rating scale can be regarded as a ‘success’ for a treatment that is able to halt disease progression. The placebo effect in people with this disease is known to be relatively high, therefore the null hypothesis response rate was set to the observed placebo effect in previous ALS trials (30%). The observed success rates with currently used treatments in neuropsychiatric conditions vary enormously. For example, the response rate to antidepressants (cumulative effect of different drugs) varies from 70-90%^5^, while even the advent of multiple anti-epileptic drugs does not reduce the rate of treatment resistant epilepsy below 15-30%^7^. Therefore, we have designed the expected success rate of ILB® in this phase II single arm trial to be 70%. This expectation will allow the differentiation of a true response from the placebo effect, and will match the expected response rate in other neuropsychiatric diseases without overinflating the expectations.

Fleming’s two-stage design^2^ was used (software made available by the University of North Carolina at Chapel Hill, <http://cancer.unc.edu/biostatistics/program/ivanova/FlemingsTwoStageDesign.aspx> ) with the following assumptions: the null hypothesis that the response rate is 0.3 (based on the observed placebo effect in ALS trials). The drug will be regarded as ‘good’ if the desirable effect (positive response = at least no decline in ALSFRS score) is observed in at least 70% of the patients (see explanations above). We expect a type I error rate of 0.05 and 80% power when the positive response rate is 0.7. The results of the analysis are summarised in Table S1 below.

**Table S1**. Exploration of trial design and power calculations. Where n is the total number of subjects; n_1_ is the number of subjects accrued during stage 1; a_1_, if a_1_ or fewer responses are observed during stage 1, the trial is stopped early for futility; b_1_, if b_1_ or more responses are observed during stage 1, the trial is stopped early and H0 (null hypothesis) is rejected; a_2_, if a_2_ or fewer responses are observed by the end of stage two, then no further investigation of the drug is warranted; b_2_, b_2_ = a_2_ + 1, if b_2_ or more responses are observed by the end of stage two, H0 is rejected; EN_0_ is the expected sample size for the trial when response rate is 0.3; EN_1_ is the expected sample size for the trial when response rate is 0.7.

| **n** | **n_1_** | **a_1_** | **b_1_** | **a_2_** | **b_2_** | **Type 1 Error** | **Power** | **EN_0_** | **EN_1_** | **Comment** |
| --- | --- | --- | --- | --- | --- | --- | --- | --- | --- | --- |
| 10 | 7 | 3 | 5 | 5 | 6 | 0.0498 | 0.825 | 7.2917 | 7.6807 | Minimax |
| 10 | 6 | 2 | 5 | 5 | 6 | 0.0472 | 0.8379 | 6.979 | 8.0374 | Minimax |
| 10 | 7 | 3 | 5 | 5 | 6 | 0.0498 | 0.825 | 7.2917 | 7.6807 | Minimax |
| 11 | 5 | 1 | 4 | 6 | 7 | 0.0435 | 0.8136 | 7.646 | 7.646 | Optimal |
| 13 | 5 | 2 | 5 | 6 | 7 | 0.0408 | 0.815 | 6.2852 | 10.3508 |  |
| 14 | 5 | 2 | 4 | 7 | 8 | 0.0439 | 0.8064 | 6.1907 | 7.7783 | Optimal |
| 14 | 5 | 2 | 4 | 7 | 8 | 0.0439 | 0.8064 | 6.1907 | 7.7783 | Optimal |

From Table S1 above it is clear that a trial design including a total of 15 patients is adequate to test the possible efficacy of the drug. It is also clear that stopping the trial early, when 13 patients were recruited did not affect the power of the trial.

Using the BOP2: Bayesian Optimal Phase II Design software (available from the University of The University of Texas MD Anderson Cancer Center: <https://www.trialdesign.org/one-page-shell.html#BOP2>^3^) we have checked the validity of our design with one interim analysis allowed. The expectations for efficacy outcome were the same as that described above (placebo effect: 30% positive outcome; accepted ‘good’ drug effect: 70% positive outcome).

Additionally, the accepted maximum rate for SAE was 5% (observed with Riluzol^8^ for the null hypothesis. The expectation was that with ILB^®^ we have no SAE (the stated stopping criteria in the protocol was one patient with a SAE possibly related to the drug). We also assume that the efficacy and toxicity of the drug are not related and expect a type I error rate at or below 0.05. With these assumptions and with a maximum of 13 patients enrolled, the results of the 10000 simulations are shown in Table S2 below. The power of this trial would be: 0.814.

**Table S2.** BOP2: Bayesian Optimal Phase II Design with Simple and Complex Endpoints

BOP2 PID: 960; Version: V1.4.10.0; Last Updated: 8/24/2021 (<https://www.trialdesign.org/one-page-shell.html#BOP2>, used on 07/12/2021).

| **Number of patients treated** | **Stop if # response <=** | **OR # toxicity >=** |
| --- | --- | --- |
| 7 | 2 | 1 |
| 13 | 6 | 1 |

The analysis would indicate that the empirically chosen sample size (with reasonable expectations based on existing knowledge at the time) would have been deemed adequate for a phase II trial.

**These estimates were produced at the request of reviewers and the editor and are not meant to be a post hoc power analysis based on the outcome of the trial^9^.**

**References**

*^1^An-Wen Chan et al., SPIRIT 2013 explanation and elaboration: guidance for protocols of clinical trials. BMJ. 2013;346:e7586.* [*https://doi.org/10.1136/bmj.e7586*](https://doi.org/10.1136/bmj.e7586)

*^2^ Jung et al., Admissible two-stage designs for phase II cancer clinical trials. Statist. Med. 2004; 23:561–569.* [*https://doi.org/10.1002/sim.1600*](https://doi.org/10.1002/sim.1600)

*^3^Lin et al., BOIN12: Bayesian optimal interval phase I/II trial design for utility-based dose finding in immunotherapy and targeted therapies. JCO Precis Oncol. 2020; 4:PO.20.00257.* [*https://doi.org/10.1200/PO.20.00257*](doi:%2010.1200/PO.20.00257)

^4^Oxnard et al., Response rate as a regulatory end point in single-arm studies of advanced solid tumors. JAMA Oncology, (2016). 2(6), 772–779. https://doi.org/[10.1001/jamaoncol.2015.6315](https://doi.org/10.1001/jamaoncol.2015.6315)

^5^Macher & Crocq, Treatment goals: response and nonresponse. Dialogues Clin Neurosci. 2004; 6(1):83–91. [*https://doi.org/10.31887/DCNS.2004.6.1/jpmacher2*](https://doi.org/10.31887/DCNS.2004.6.1/jpmacher2)

^6^Vickers et al., Setting the bar in phase II trials: the use of historical data for determining "go/no go" decision for definitive phase III testing. Clin Cancer Res. 2007;13(3):972–976. [*https://doi.org/10.1158/1078-0432.CCR-06-0909*](https://doi.org/10.1158/1078-0432.CCR-06-0909)

^7^Picot et al., *The prevalence of epilepsy and pharmacoresistant epilepsy in adults: a population-based study in a Western European country. Epilepsia. 2008; 49(7):1230-8.* [*https://doi.org/10.1111/j.1528-1167.2008.01579.x*](https://doi.org/10.1111/j.1528-1167.2008.01579.x)*.*

*^8^Bensimon et al., Riluzole treatment, survival and diagnostic criteria in Parkinson plus disorders: The NNIPPS Study. Brain 2009; 132:156–171.* [*https://doi.org/10.1093/brain/awn291*](https://doi.org/10.1093/brain/awn291)

*^9^Zhang et al., Post hoc power analysis: is it an informative and meaningful analysis? Zhang Y, et al. General Psychiatry 2019;32:e100069.* [*http://dx.doi.org/10.1136/gpsych-2019-100069*](http://dx.doi.org/10.1136/gpsych-2019-100069)
