# Supplementary material for: A phase II open label clinical study of the safety, tolerability and efficacy of ILB® for Amyotrophic Lateral Sclerosis
Source: PLoS One. 2022 May 25;17(5):e0267183. doi: 10.1371/journal.pone.0267183 (PMC9132272; doi:10.1371/journal.pone.0267183)
Supplement: Attachment — Submitted filename: PONE-D-21-04468, entitled A phase II open label clinical study of the safety, tolerability and efficacy of a low molecular weight dextran sulphate for Amyotrophic Lateral Sclerosis.docx [file pone.0267183.s004.docx]

Ann Logan et al.

Amyotrophic lateral sclerosis (ALS) is a neurodegenerative disorder characterised by rapid progression of upper and lower motor neuron loss, which causes paralysis and death within 2-5 years from disease onset. However only riluzole, which have beneficial effects in a limited population of patients with ALS, have been approved.

In this manuscript, Ann Logan et al., revealed that no serious adverse events or participant withdrawal were observed in clinical trial of LMW-DS in patients with ALS. The results also showed that ALSFRS-R improved after treatment. Although this study provides important results, this paper will be improved by addressing these issues. The specific concerns are listed below.

Major issues:

1. Progression rate of ALSFRS-R and disease duration should be included in the background of patients, as the change in ALSFRS-R is strongly influenced by the disease progression.
2. Effectiveness of treatment should be established by the demonstration of a treatment effect on function in daily activities such as ALFRS-R. However, given the open-label trial, it would have been helpful to include quantitative motor function as an objective measurement.
3. It is not a scientific expression to state that a patient's vitality has increased, especially in an open-label trial.
4. FDA recommends the consideration of add-on designs, in which a treatment previously shown to be effective for the treatment of ALS is given to all patients participating in the trial, with patients randomized to the added investigational drug or added placebo. Too much justification for open-label trial should be avoided. In addition, it should be explained in detail that patients taking riluzole are not eligible to participate in this trial.
5. Pain and autonomic dysfunction are not typical neurological findings in ALS patients, why did you assess these symptoms?
6. I can't find out the details of the scores for severity of the autonomic and sensory symptoms, but are vertigo and fatigue classified autonomic or sensory symptoms?
7. To investigate the correlation between the change of serum HGF and clinical measures such as ALSFRS-R would support the hypothesis that increased serum HGF improves motor function of patients with ALS.

Minor issues

1. What subscores of the ALSFRS-R have improved in ALS patients?
2. HGF should be spelled out.
3. The authors need to correct writing errors (FCV→FVC, Line226; forced ventilatory capacity→forced vital capacity, Line294).
