# Supplementary material for: A phase II open label clinical study of the safety, tolerability and efficacy of ILB® for Amyotrophic Lateral Sclerosis
Source: PLoS One. 2022 May 25;17(5):e0267183. doi: 10.1371/journal.pone.0267183 (PMC9132272; doi:10.1371/journal.pone.0267183)
Supplement: Attachment — Submitted filename: Response to Reviewers.docx [file pone.0267183.s005.docx]

*Ann Logan et al.* A phase II open label clinical study of the safety, tolerability and efficacy of ILB^®^ for Amyotrophic Lateral Sclerosis

**Response to Editor and Reviewers**

We appreciate the detailed and helpful comments of the reviewers that we have considered carefully, and we believe that the revisions we have made in response have improved the manuscript significantly.

Please note that we have replaced the Recommended International Non-Proprietary Name (rINN) for the drug under test with its proprietary name and defined the rINN in the abstract, introduction and methods sections.

**Editor**

*Major issues:*

1. Progression rate of ALSFRS-R and disease duration should be included in the background of patients, as the change in ALSFRS-R is strongly influenced by the disease progression.

***Response:*** *The Lead Clinical Investigator for this trial has treated more than 1000 ALS patients during his 45 years as a neurologist, following patients from diagnosis until death and has made multiple studies both on drug treatment as well as on the course of the disease. In Scandinavia there are at least 30 different ALS forms, by genetic, inflammatory and/or destructive kind. ALS is thus a very heterogeneous constellation of disease. ALS causes a degeneration of neurons and glia. The destruction of cells and the progress of disease is not linear in each patient during his/her course of disease: some months they have a slow decrease in function and during other periods they have a more rapid progress. There can be a variable arrest of symptoms for months or even years in some patients, but the survival from first symptoms till death is usually 2-5 years, while 10 % have a longer survival. The disease appears in 65% of males, in 35 % of females. The course is often slower in females than in males.*

*Together, this makes pre-treatment disease progression and disease duration of very limited predictive value in indicating response to treatment of this very heterogeneous disease. Indeed, this small pilot safety study was open to patients of a deliberately heterogeneous background to ensure the relevance of the outcomes to most ALS etiologies.* *The patients were selected to have different clinical characteristics with respect to the mixture of rate of progress, of age and sex, the type of dysfunction (genetic or sporadic disease, flaccid or spastic paresis, talking or swallowing problems and rate of muscle weakness/muscle atrophy/grade of spasticity).*

*What is beyond dispute is that, whatever the etiology and clinical characteristics of the patient, once ALS is diagnosed disease progression is NEVER REVERSED so ANY indication of such a response in the outcome data is important. As this small pilot study was designed primarily as a safety and tolerability study for a heterogeneous patient cohort we have, accordingly, been* ***very cautious*** *in attaching significance to the observations of changes in the ALSFRS-R scores despite a positive response from all treated patients.*

*Notwithstanding the above discussion, as requested we have now included data on the ‘Years since onset of disease symptoms’ for each patient in a* ***new Table 4 on Page 23/24*** *in the Results section (necessitating renumbering of all Tables).*

2. Effectiveness of treatment should be established by the demonstration of a treatment effect on function in daily activities such as ALFRS-R. However, given the open-label trial, it would have been helpful to include quantitative motor function as an objective measurement.

***Response:*** *Quantifiable muscle strength and muscle enzymes depend on the amount of muscle exercise achieved by individuals from day to day, this varies from day to day and patient to patient. Some ALS patients do not even have major peripheral muscle dysfunction but have swallowing or spasticity problems dominating. Most of the challenges in quantitating motor function in ALS patients therefore relate to the disease’s remarkably protean nature. Such heterogeneity adds a challenge that is not present for many other neuromuscular conditions. The difficulties in assessing motor function in ALS patients are described in detail in this review: Rutkove, S.B. Clinical Measures of Disease Progression in Amyotrophic Lateral Sclerosis. Neurotherapeutics* ***12,*** *384–393 (2015).* [*https://doi.org/10.1007/s13311-014-0331-9*](https://doi.org/10.1007/s13311-014-0331-9)

*We have used two of the most reliable and widely used clinical rating scales for ALS in this study: the ALSFRS-R, which is simple to use in patients with muscle and swallowing problems, and the Norris scale, which is more detailed and elucidates a more comprehensive picture of patient functions. Of relevance here, and described on* ***Page 36 Lines 623-625****, many of the assessments made as part of ALSFRS-R and Norris (both are Functional Rating Scores) relate to both gross and fine motor skills, e.g., handwriting, speech, swallowing, respiration, dressing, walking, turning in bed, cutting food, climbing stairs, etc., and the scores in these categories show close agreement with objective measures of muscle strength (reference 30).*

*It should also be noted that this is the first early and small initial safety study of the ILB^®^ in ALS and a more comprehensive testing regime can be incorporated into future larger scale placebo-controlled trials with efficacy as a primary outcome.*

3. It is not a scientific expression to state that a patient's vitality has increased, especially in an open-label trial.

***Response:*** *Vitality is related to the patient’s perceived levels of energy and fatigue. This was specifically assessed and subjectively quantified as part of the sensory and autonomic symptom panel (tiredness and fatigue subscores). When the scores for these categories were compared before and after treatment, there was a decrease of 25% (P<0.02). This has now been better defined and clarified on* ***Page 35 Lines 600-610****.*

4. FDA recommends the consideration of add-on designs, in which a treatment previously shown to be effective for the treatment of ALS is given to all patients participating in the trial, with patients randomized to the added investigational drug or added placebo. Too much justification for open-label trial should be avoided.

***Response****: We believe that detailing the steps we have taken in the trial design to ensure the generation of meaningful proof of concept data from a small patient cohort are important to those readers who may not be fully cognisant of the challenges in developing drugs for rare orphan diseases with fatal outcomes. However, we accept that we may have laboured this point somewhat. Accordingly, we have shortened this first paragraph in the discussion on* ***Pages 32-33*** ***Lines 541-548*** *to make it more specific.*

5. In addition, it should be explained in detail that patients taking riluzole are not eligible to participate in this trial.

***Response****:* *Motor neuron dysfunction and degeneration in ALS involves several pathogenic mechanisms, which include disturbed energy metabolism, cytoskeletal abnormalities, changes in transcription, glutamate excitotoxicity, glial hyperactivation and reduced glutamate uptake. In our in vitro models we found that the ILB^®^ is able to modulate molecular processes relevant to ALS by modulating the biological effects of growth factors, with multiple downstream consequences. In glial cells the ILB^®^ reduces oxidative stress, mitochondrial dysfunction and glial activation. It also promotes cell survival, differentiation, MBP expression and glutamate uptake. In neuronal cultures ILB^®^ reduces neuronal cell death, oxidative stress and mitochondrial dysfunction. In neurons the compound also promotes differentiation, neurite outgrowth and cellular homeostasis.* *Most importantly glutamate production and release in neuronal cells is NOT affected by ILB^®^. Rather, ILB^®^ enhances glutamate uptake by astrocytes. Glutamate production and release (followed by Ca+-mediated signalling cascades) in neuronal cells is one of the most important factors required for synaptic remodelling necessary for regeneration and repair of neuronal networks affected in ALS (and indeed all neurodegenerative diseases). This process is controlled by the growth factors modulated by ILB^®^ and leads to neurite outgrowth and differentiation of these cells. This is the process that will lead to a measurable functional benefit in the nervous system and clinical improvement in the patients with ALS.*

*Riluzole and lamotrigine are drugs that affect several neurotransmitter systems and signalling pathways (see references 12,13 and 14 in the manuscript). In vitro studies and patients side effect profiles of the drug demonstrate that riluzole/lamotrigine affects glutamatergic signalling in neurones and most importantly inhibit Ca^+^-dependent downstream signalling in these cells. This means that riluzole and lamotrigine inhibit some of the same mechanisms that are pivotal for the restoration of neural function induced by ILB^®^. In summary, based on the established mechanism of action of our drug, it is clear that riluzole/lamotrigine will interfere with key ILB^®^ actions and prevent the beneficial effects in patients.*

*As requested, this has now been set out in detail on* ***Pages 8-9*** ***Lines 164-195*** *of the revised manuscript.*

6. Pain and autonomic dysfunction are not typical neurological findings in ALS patients, why did you assess these symptoms?

***Response:*** *In fact, 30-50 % of ALS patients have pain and other sensory and autonomic symptoms and dysfunction during the course of their ALS disease.* *This is well described in the following review and editorial:*

*Chiò A, Mora G, Lauria G. Pain in amyotrophic lateral sclerosis. Lancet Neurol. 2017 Feb;16(2):144-157. doi: 10.1016/S1474-4422(16)30358-1. Epub 2016 Dec 8. PMID: 27964824.*

*Vucic S. Sensory and autonomic nervous system dysfunction in amyotrophic lateral sclerosis. Neuropathol Appl Neurobiol. 2017 Feb;43(2):99-101. doi: 10.1111/nan.12336. PMID: 27333192.*

*The subjective occurrence of pain, sensory and autonomic complaints and symptoms are easily rated as we have done in this study and this monitoring is important to the patients and next of kin. The information is used to inform treatment options for individual patients. To clarify for the reader, we have re-named these symptoms as* ***Subjective Sensory and Autonomic Symptoms on Page 20 Line330****.*

*Below are some of our publications that describe the impact of these symptoms and our contribution to their investigation:*

- *Olsson AG, Graneheim Hellgren U, Persson LI, Strand S. Factors that facilitate and hinder the manageability of living with amyotrophic lateral sclerosis in both patients and next of kin. Palliative Support Care 2013;11(3):183-9*
- *Olsson, AG, Markhede I, Strand S, Persson LI. Well-being in patients with amyotrophic lateral sclerosis and their next of kin over time. Acta Neurol Scand 2010;121:244-250.*
- *Olsson AG, Markhede I, Strang S, Persson LI. Differences in quality of life modalities give rise to needs of individual support in patients with ALS and their next of kin. Palliative Support Care 2010; 8:75-86.*

7. I can't find out the details of the scores for severity of the autonomic and sensory symptoms, but are vertigo and fatigue classified autonomic or sensory symptoms?

***Response****:* *Vertigo is often a symptom of an autonomic neuropathy but may also have a sensory causation. Similarly, fatigue is strongly associated with autonomic dysfunction.*

*The 16 symptom categories related to autonomic/sensory dysfunction that were evaluated in this study were as follows:* *pain; paresthesia; coldness; sweating; constipation; frequency of faeces; frequency of urination; urination difficulty, numbness; fatigue; tiredness; insomnia; arrhythmia; tachycardia; frequent waking; vertigo/dizziness.*

*This is now better detailed in the Methods section now entitled Subjective Rating of Sensory and Autonomic Symptoms on* ***Page 20-21, Lines 334-342****.*

8. To investigate the correlation between the change of serum HGF and clinical measures such as ALSFRS-R would support the hypothesis that increased serum HGF improves motor function of patients with ALS.

***Response:*** *This small study has not generated data that can establish a causal relationship between HGF levels and change in ALSFRS-R scores. We note that clinical trials delivering recombinant HGF have achieved serum levels of 20,000 pg/mL with measurable functional effects (e.g., Ido A, Moriuchi A, Numata M, Murayama T, Teramukai S, Marusawa H, Yamaji N, Setoyama H, Kim ID, Chiba T, Higuchi S, Yokode M, Fukushima M, Shimizu A, Tsubouchi H. Safety and pharmacokinetics of recombinant human hepatocyte growth factor (rh-HGF) in patients with fulminant hepatitis: a phase I/II clinical trial, following preclinical studies to ensure safety. J Transl Med. 2011 May 8;9:55. doi: 10.1186/1479-5876-9-55. PMID: 21548996; PMCID: PMC3112439). Our observation that ILB^®^ treatment elevates serum levels to 41,691 pg/mL suggest that the HGF levels achieved in this cohort of patients with ALS are more than sufficient to activate downstream cellular signalling through the C-Met receptor, with beneficial functional effects as indicated. Larger studies with variable doses of ILB^®^ versus placebo lie in the future and these may allow such a link to be made.*

*Minor issues*

9. What subscores of the ALSFRS-R have improved in ALS patients?

***Response****: Since this cohort of patients was deliberately chosen to be heterogeneous, their functional insufficiencies were also heterogeneous and varied from individual to individual. Accordingly, when the patient data was pooled we saw improvements in all ALSFRS-R subscores, but the extent of improvement in each category varied between individuals. It is therefore meaningless to analyse and compare individual ALSFRS-R subscores in this small cohort of patients.*

10. HGF should be spelled out.

***Response****: We have done this on* ***Page 5 Line 98-99****.*

11. The authors need to correct writing errors (FCV→FVC, Line226; forced ventilatory capacity→forced vital capacity, Line 294).

***Response****:* We have corrected this on **Pages 18 Line 272-273 and Page 21 Line 344.**

**Reviewer 1**

*Major issues:*
1.	Progression rate of ALSFRS-R and disease duration should be included in the background of patients, as the change in ALSFRS-R is strongly influenced by the disease progression.

***Response:*** *See response to Editor Point 1.*

2.	Effectiveness of treatment should be established by the demonstration of a treatment effect on function in daily activities such as ALFRS-R. However, given the open-label trial, it would have been helpful to include quantitative motor function as an objective measurement.

***Response****: See response to Editor Point 2.*

3.	It is not a scientific expression to state that a patient's vitality has increased, especially in an open-label trial.

***Response:*** *See response to Editor Point 3.*

4.	FDA recommends the consideration of add-on designs, in which a treatment previously shown to be effective for the treatment of ALS is given to all patients participating in the trial, with patients randomized to the added investigational drug or added placebo. Too much justification for open-label trial should be avoided. In addition, it should be explained in detail that patients taking riluzole are not eligible to participate in this trial.

*Response: See response to Editor Point 4 and 5.*

5.	Pain and autonomic dysfunction are not typical neurological findings in ALS patients, why did you assess these symptoms?

*Response: See response to Editor Point 6.*

6.	I can't find out the details of the scores for severity of the autonomic and sensory symptoms, but are vertigo and fatigue classified autonomic or sensory symptoms?

***Response:*** *See response to Editor Point 7.*

7.	To investigate the correlation between the change of serum HGF and clinical measures such as ALSFRS-R would support the hypothesis that increased serum HGF improves motor function of patients with ALS.
***Response:*** *See response to Editor Point 8.*

*Minor issues*
8.	What subscores of the ALSFRS-R have improved in ALS patients?

***Response:*** *See response to Editor Point 9.*

9.	HGF should be spelled out.

***Response:*** *See response to Editor Point 10.*

10.The authors need to correct writing errors (FCV→FVC, Line226; forced ventilatory capacity→forced vital capacity, Line294).

***Response:*** *See response to Editor Point 11.*

**Reviewer 2**

1. This is a manuscript reporting the outcome in an early Phase 2 trial. As it stands there are serious issues in the reporting as the paper does not reflect the protocol. For example, the primary endpoints of the protocol are not mentioned here; nor are the success criteria - what is considered acceptable on these endpoints?

***Response:*** *The primary and secondary endpoints of the trial were clearly stated on* ***Page 13 Lines 213-215*** *of the manuscript* *as being ‘drug safety and tolerability, secondary endpoints relating to drug efficacy and mechanism of action were also included’. These primary and secondary outcomes were also described in more detail in* ***Section 7 of the supplementary document S1 Trial Protocol,*** *in which* ***Appendix One*** *defines the specific risk assessments made and the treatment stopping criteria as* *‘clinical findings which constitute a medical significant risk’. This explicitly states this to be defined as: ‘Serious Adverse Events (SAE) that are assessed as possibly or probably related to study treatment, including all risks categorized as of grade C (as stated in Appendix 1).*

*As described in the European Medicines Agency document entitled* ***ICH Topic E 9 Statistical Principles for Clinical Trials****: ‘In all clinical trials evaluation of safety and tolerability constitutes an important element. In early phases this evaluation is mostly of an exploratory nature, and is only sensitive to frank expressions of toxicity, whereas in later phases the establishment of the safety and tolerability profile of a drug can be characterised more fully in larger samples of subjects’. Accordingly, this small pilot trial gathered data on all adverse effects off the drug and was specifically looking for drug-related SAE.* *The absence of any drug-related SAE and the tolerability of the drug were deemed to be the primary criteria for success in this study.*

*In response to the reviewer, we have now added a sentence to the methods section of manuscript on* ***Page 13*** ***Lines 215-216*** *that describe what we deemed to be appropriate success criteria for these primary outcomes. We have also added a related comment in the Discussion on* ***Page 33 Lines 564-567.***

2.	Also the trial was stated to recruit 15 patients. Only 13 were apparently approached according to the flow chart (not a CONSORT diagram as the trial is not randomised). So why was the number to be approached changed? Why was the trial not conducted as stated in the protocol?

***Response:*** *Note that we used the 2016 CONSORT diagram relevant to pilot and feasibility trials in Figure 1 with adaptions as recommended by Lancaster, G.A., Thabane, L. Guidelines for reporting non-randomised pilot and feasibility studies. Pilot Feasibility Stud* ***5,*** *114 (2019).* [*https://doi.org/10.1186/s40814-019-0499-1*](https://doi.org/10.1186/s40814-019-0499-1)*.*

*The planned number of patients to be included in this safety and tolerability study was 15, however due to delays in recruitment, and the fact that no SAEs or other safety concerns were reported in the first 13 patients, it was decided by the Sponsor, along with the local clinical and ethical agencies, to terminate patient recruitment and conclude the study with the 13.*

*The intention to widen this first safety study to a conventional placebo-controlled study was always planned and, as the findings at this first pilot study indicated safety, tolerability and a positive clinical response in all of the first 13 patients tested, it was decided to shorten the study from the planned 15 to 13 patients, to save 5 months of study time, complete the data analysis of the first 13 patients, and use this to more rapidly initiate planning of a larger conventional placebo-controlled study.*

*This change in recruitment number plus the reason for it is now described in the Results section on* ***Page 22 Lines 382-87****.*

3.	The methods section does not state the aspiration of the study, and the actual planned number is hidden in the protocol.
***Response:*** *We clearly stated the planned primary outcomes (aspirations?) of this trial in the methods on* ***Page 6 Lines 113-114*** *and again on* ***Page 13 Lines 213-215*** *in the Methods section. We also mention this in the Abstract* ***(Page 3 Lines 32-34)****, state a hypothesis in the last paragraph of the Introduction (****Page 6 Lines 103-106****) and I quote from* ***Page 6 Lines 112-114****: ‘The clinical trial was a Phase IIa, single-centre, open label, single-arm proof of concept study of a small number of patients, where the primary endpoint was safety and tolerability of subcutaneously administered ILB®.’*

*As requested, we have now described the planned versus actual number of patients recruited to the trial, plus the reason for the change, on* ***Page 22 Lines 382-387*** *in the Results section.*

4.	The patient characteristics are a result and not a method - this section including Table 1 needs to lead the results.
***Response:*** *As the reviewer suggests we have moved this information to a new Results section entitled ‘Patient demographics and baseline clinical characteristics with renumbered Tables 3 and 4 on* ***Page 23-24.***

5.	Please explain why the use of statistical tests here is not in line with the protocol which talks of summary measures only. Please give actual p-values and not just p<0.xx when the p-value exceeds 0.001. What work was done to demonstrate that the data were Normally distributed?
***Response****: As is well established, the summary measures indicated in the trial protocol provide a quick and simple description of the data and include mean, median, mode, minimum value, maximum value, range, standard deviation, etc. It is appropriate to use this data to perform a paired Student t test or ANOVA to compare the mean of two groups of related samples and to evaluate whether the means of the two sets of data are statistically significantly different from each other. These analyses are described in the Statistical Analysis Plan associated with the clinical trial protocol (see Appendix S1). Exact P values have been given (for example, see Table 8* ***on Page 31****). Normal distribution of data was confirmed by comparing medians and means and by plotting histograms of the frequency distribution of the analysis variable.*

6.	It is incorrect to only report significant results (e.g., Table 7) - please give effect sizes and CI here and report all tests done - what was the hypothesis here?

***Response:*** *The trial protocol and the methods section clearly describes all of the measurements taken on these patients and the related raw data is available on-line to the interested reader who wants more in depth information (link provided in the manuscript on* ***Page 26 Lines 442-443*** *at* <https://www.clinicaltrialsregister.eu/ctr-search/trial/2017-005065-47/results> and available on request from the authors)*. To report all data pertaining to non-significant differences within the manuscript would seem superfluous and of limited value to most readers.*

*The trial hypothesis is clearly stated on* ***Page 5 Lines 103-106*** *of the Introduction.*

7.	Dynamite plots (e.g. Fig 4,5) give less information than a table would - especially given the serial nature of the data. Figure 5C is in particular the wrong figure and again selects only the significant differences.

***Response:*** *We respectfully disagree since the source data is available for detailed evaluation by the interested reader on-line at:* [*https://www.clinicaltrialsregister.eu/ctr-search/trial/2017-005065-47/results*](https://www.clinicaltrialsregister.eu/ctr-search/trial/2017-005065-47/results)

*and is also available on request from the authors. We feel that the graphical representation of the data we have made illustrate the outcomes in a visual and easily understandable way, particularly for the non-expert reader.*

8	Figure 6 would be clearer as a table.

***Response:*** *We respectfully suggest that the graphical representation of the data we have made in Figure 6 better illustrates the changes in clinical characteristics before and after treatment than would a large table of numbers.*

**Reviewer 3**

1. This is a concept-of-proof open, uncontrolled study on a very small and clinically heterogeneous ALS population treated with ILB® and followed up for a short time. The molecule is supposed to be effective mainly by stimulating the production of HGF. The molecule and hypothesized mechanism of action is quite innovative and interesting. In addition, new trials in this population are always welcome given the lack of effective treatments for such a severe and incurable condition. Nevertheless, preclinical data about ILB® in neurological disorders are limited and experiments on ALS animal models are lacking. This limitation must be declared by the authors.

***Response:*** *As requested, we have added a short section in the Introduction on* ***Page 5*** ***Lines 93-97*** *to address this limitation.*

2.	Safety and tolerability as primary endpoint are well documented and particular attention has been paid to any coagulation disorder potentially related to HGF. In my view, nevertheless, no inference can be drawn about clinical advantages. A clinical impression of "increased vitality, decreased spasticity and increased mobility" and even a transient improvement in functional scales have no relevance in such a small uncontrolled trial. Only robust changes in biological markers can be taken into account in this phase of the experimental study. This strong limitation must be stressed by the authors.

***Response:*** *We believe that we have been very cautious in making any claims about the efficacy of ILB^®^, focussing primarily on safety and tolerability and only indicating potential for efficacy. Indeed, we state in the discussion about efficacy on* ***Page 34 Line 586-590****: ‘Although the small size and open-label nature of this study, as well as the lack of rigour of the statistical analysis, necessitates an* ***extremely cautious interpretation****, the results of the secondary endpoints (including a limited panel of blood biomarkers that are reported here and elsewhere [39]) suggest the potential for treatment-related change in the progression of ALS.’*

*Further evidence of drug efficacy in this same cohort of patients is provided by the serum biomarker results (myoglobin and creatine kinase levels reported here and levels of metabolic biomarkers now reported elsewhere: Lazzarino G****,*** *Mangione R****,*** *Belli A****,*** *Di Pietro V****,*** *Nagy Z, Barnes NM****,*** *Bruce L****,*** *Ropero BM****,*** *Persson LI****,*** *Manca B****,*** *Tavazzi B****,*** *Lazzarino G, Logan A. ILB^®^ attenuates oxidative/nitrosative stress and mitochondrial dysfunction in patients with Amyotrophic Lateral Sclerosis. Journal of Personlized Medicine 2021, 11, x. Available from:* [*https://doi.org/10.3390/xxxxx*](https://doi.org/10.3390/xxxxx) *In press).*

*As requested, we have added this reference in the Discussion section [new Reference 39] to indicate the biomarker data generated in this patient cohort and also emphasised this limitation in the Discussion on* ***Page 34*** ***Lines 586-590****.*

**Reviewer 4**

1.	ALSFRS-R score improves on average of 4 points after 3 months since the first injection of the IMP (and after 2 months after the last injection). Then, how do the authors explain the worsening of FVC values at day 99? Which are the ALSFRS-R items improving?

***Response:*** *The FVC values decline at Day 99 along with a resumption of decline in ALSFRS-R and Norris scores at Day 99. As described, ILB^®^ treatment seems to have slowed, but not reversed, the decline in FVC values, presumably because of the more muted response of the complex neuromuscular control of this function. This attenuation in decline of the FVC values is also reflected in the lack of change in the ALSFRS-R sub-score for Respiratory Insufficiency. Please also see Point 9 of my response to the Editor.*

2.	What is the reference interval for CK values? why there was no blood CK sampling at day 99? Are creatinine values available?

***Response****: we apologise as we mistakenly defined the creatine kinase units as U/L in old Table 7 when the assay actually measured plasma creatine kinase as ukat/L. We have corrected this by converting the measured ukat/L values of creatine kinase to U/L in new Table 8 on* ***Page 31*** *and this reveals the change from 421±337 U/L at Day 1 (Pre-treatment) to 364±299 U/L at Day 36 (Post-treatment). In a healthy Swedish adults, the serum CK level is reported to vary with a number of factors (gender, race and activity), but the normal range is 24-204* *U/L (the reference interval) and healthy adult values of CK are reported in Reference 41. Higher amounts of serum CK can indicate muscle damage due to chronic disease or acute muscle injury (see Reference 41).*

*The protocol indicates that no blood samples were taken for biomarkers at Day 99 in order to minimise interventions on this very sick cohort of patients. Creatine levels were measured in these patients but there was no change in the values recorded over the monitoring period (reported in new Reference 39).*

3.	Why did the authors evaluate autonomic functions? What exactly is the autonomic and sensory rating scale used in the trial?
***Response:*** *See Points 6 and 7 of the response to the Editor.*

4.	The IMP used in this study seems to reverse the natural decline of ALSFRS-R overtime observed in ALS (without affecting objective measures like NFL or FVC). It is established that NFLs are stable throughout the disease, therefore NFL stabilization shown here was expected and it is not a "positive" result. Furthermore, benefit is prompt. Authors should discuss, somewhere in the discussion, the chance of a placebo effect.

***Response:*** *The senior Clinical Investigator in this clinical trial has worked over many years with the biochemist (nowadays a neurologist) Lars Rosengren on studies of CSF biomarkers in ALS and, with others, they have found that the progress of disease in ALS is parallel to CSF and blood levels of Neurofilament Light Chain. Please see References 4 and 5 as examples. In addition to the work of the Clinical Investigator, there are numerous other publications describing the diagnostic and prognostic value of NfL in ALS (for example, see the review by Poesen K, Van Damme P. Diagnostic and Prognostic Performance of Neurofilaments in ALS. Front Neurol. 2019 Jan 18;9:1167. doi: 10.3389/fneur.2018.01167. PMID: 30713520; PMCID: PMC6345692). These publications all describe how the levels of NfL correlate with parameters of disease severity, such as the decline in the ALSFRS-R, and can be used to monitor disease progression. Hence, stabilization of NfL levels may be indicative of a ‘positive’ result.*

*A placebo group is used in randomised controlled trials to control for the placebo effect and to more accurately determine the efficacy of a new treatment. However, it is also well known that a placebo might also be associated with negative effects (the nocebo effect). Interestingly, a strong nocebo effect has been noted in trials of drugs to treat patients with amyotrophic lateral sclerosis, most recently reported in trials evaluating riluzole and edaravone, drugs which have a partially overlapping mechanism of action to ILB® evaluated here. For unknown reasons, the nocebo effect seems to be stronger in patients with amyotrophic lateral sclerosis than in other neurological disorders (see Rato ML, Duarte GS, Mestre T, de Carvalho M, Ferreira JJ. Strong nocebo effects in amyotrophic lateral sclerosis trials might mask conclusions. Lancet Neurol. 2018 Oct;17(10):842. doi: 10.1016/S1474-4422(18)30310-7. Epub 2018 Sep 18. PMID: 30264722) which could, in theory, mask the beneficial effects of ILB^®^. As the reviewer suggests, we have included a short section on potential placebo effects in patients with ALS in the Discussion on* ***Pages*** ***34-35 Lines 590-595.***

5.	Accordingly, why did not the authors evaluate QoL at day 99?

***Response:*** *The trial protocol indicates that QoL were not evaluated at Day 99 in order to minimise the research burden on this very sick cohort of patients.*
