# Supplementary material for: A phase II open label clinical study of the safety, tolerability and efficacy of ILB® for Amyotrophic Lateral Sclerosis
Source: PLoS One. 2022 May 25;17(5):e0267183. doi: 10.1371/journal.pone.0267183 (PMC9132272; doi:10.1371/journal.pone.0267183)
Supplement: Attachment — Submitted filename: Revision_PONE-D-21-04468 entitled A phase II open label clinical study of the safety tolerability and efficacy of a low molecular.docx [file pone.0267183.s006.docx]

Ann Logan et al.

Given that ALS is a heterogeneous disease, as the author mentions, I am less certain than the authors in that their results indicate a positive response from all the treated patients. The revision raises some additional issues:

Major issues:

1. As the authors emphasize, ALS is a heterogeneous disease. In fact, several previous studies have shown that the progression rate of ALSFRS-R is useful in predicting disease progression. Therefore, a clear explanation why the authors did not stratify the subjects using the progression rate of ALSFRS-R is necessary.
2. Unfortunately, there is a lack of confidence in the overall ADL scores, as some subjects have an improvement in Norris score of more than 20 points despite a decrease in ALSFRS-R by more than 5 points in Fig 6. How should I interpret that?
3. If the authors ‘deliberately’ selected patients with different backgrounds as described in the response, it should be sufficiently described in the criteria for patient selection in Methods. A detailed explanation as for how the authors eliminated selection bias is needed.
4. If the ADL scale is unreliable due to the fact that it is an open-label trial and the results of quantitative motor function test do not exist, it is preferred not to mention too much about the efficacy of the drug.
5. Although dizziness and fatigue can be related to autonomic symptoms, it is not common to include them in the same category due to the wide range of mechanisms.

Minor issue:

1. In the table4, the patient's number is different from the other descriptions.
