# Supplementary material for: A phase II open label clinical study of the safety, tolerability and efficacy of ILB® for Amyotrophic Lateral Sclerosis
Source: PLoS One. 2022 May 25;17(5):e0267183. doi: 10.1371/journal.pone.0267183 (PMC9132272; doi:10.1371/journal.pone.0267183)
Supplement: Attachment — Submitted filename: Response to Reviewers .docx [file pone.0267183.s007.docx]

*Ann Logan et al.* A phase II open label clinical study of the safety, tolerability and efficacy of ILB^®^ for Amyotrophic Lateral Sclerosis

**Response to Editor and Reviewers**

We appreciate the further detailed and helpful comments of the reviewers that we have again considered carefully. We believe that the revisions we have made in response to these comments have answered their questions and improved the manuscript significantly.

**Editor**

Given that ALS is a heterogeneous disease, as the author mentions, I am less certain than the authors that their results indicate a positive response from all the treated patients.

***Response****: We have tried to be extremely cautious in our interpretation of the efficacy data throughout. In this revised version of the manuscript, we have included new data regarding the apparent effects of ILB on disease progression by comparing expected versus actual changes in ALSFRS-R scores (new Table 7). We have also emphasized the limitations of this study, the potential for placebo effects and the fact that the results only indicate ‘potential’ as a treatment for ALS (Lines 603-609 and 717-723).*

The revision raises some additional issues:

*Major issues:*

1. As the authors emphasize, ALS is a heterogeneous disease. In fact, several previous studies have shown that the progression rate of ALSFRS-R is useful in predicting disease progression. Therefore, a clear explanation why the authors did not stratify the subjects using the progression rate of ALSFRS-R is necessary.

***Response****: With the extremely small patient group stratification would not have been possible. However, as suggested we have now used the expected progression rate of the ALSFRS-R scores to expand Table 4 to provide more detail for individual patients on disease characteristics and expectations of disease progression rate during the study period (Table 4 in revised manuscript). Furthermore, we have derived the predicted ALSFRS-R score at key post-treatment intervals for each patient based on their pre-trial disease progression rates as described by Taylor et al., 2016 (new reference number 36). Table 7 now describes the predicted versus actual ALSFRS-R scores for each individual patient at Day 36 and 99, based on their pre-treatment rate of disease progression.*

*While the added data indicate that ILB treatment does indeed slow the disease progression rate in all patients, our interpretation remains very cautious due to the limitations of this small study.*

2. Unfortunately, there is a lack of confidence in the overall ADL scores, as some subjects have an improvement in Norris score of more than 20 points despite a decrease in ALSFRS-R by more than 5 points in Fig 6. How should I interpret that?

***Response****: Looking at Figure 6 it can be seen that just one patient (Patient 8) has a significant lack of correlation of the 2 scoring systems. While the Norris and ALSFRS-R scores do show a relatively good correlation, the two scoring systems reflect very different scoring parameters. Therefore, differences in the scores are not unexpected and should not be overinterpreted. Nevertheless, we have now removed Figure 6 from the manuscript and replaced it with new Table 7. As suggested by Reviewer 1 and the Editor, we have derived the predicted ALSFRS-R score at key post-treatment intervals for each patient based on their pre-trial disease progression rates as described by Taylor et al., 2016 (new reference number 36). Table 7 now describes the predicted versus actual ALSFRS-R scores for each individual patient at Day 36 and 99, based on their pre-treatment rate of disease progression. We believe that this new table better describes the changes experienced by each of the trial participants.*

3. If the authors ‘deliberately’ selected patients with different backgrounds as described in the response, it should be sufficiently described in the criteria for patient selection in Methods. A detailed explanation as for how the authors eliminated selection bias is needed.

***Response****: The inclusion/exclusion criteria used for patient selection are described in detail in Table 1 of the Methods section. The heterogenous patient group we recruited reflects the fact that we ‘deliberately’ did NOT use any enrichment methods to obtain a more homogenous patient population but used the prevalent qualifying cases available in the clinic that carried out the clinical trial.*

4. If the ADL scale is unreliable due to the fact that it is an open-label trial and the results of quantitative motor function test do not exist, it is preferred not to mention too much about the efficacy of the drug.

**Response**: The functional rating scales (ALSFRS-R and Norris) used in this trial are widely used in clinical trial settings. As mentioned previously, the open label nature of trial may indeed enhance possible placebo effects. However, placebo effects cannot explain the degree of change seen in this cohort of patients (see new reference number 41). Despite this, our interpretations regarding efficacy are very cautious, confined and tempered by the study limitations.

5. Although dizziness and fatigue can be related to autonomic symptoms, it is not common to include them in the same category due to the wide range of mechanisms.

***Response****: We have now recategorized these symptoms as non-motor, autonomic and sensory symptoms (Lines 316 and 321).*

*Minor issue:*

1. In the table 4, the patient's number is different from the other descriptions.

***Response****: We have corrected this in Table 4 of the manuscript so that patient number is consistent throughout the manuscript.*

**Reviewer 3**

1. In table 1 please specify diagnosis of ALS as "definite" to uniform it to inclusion criteria in the text.

***Response****: This has been specified in Table 1 as requested.*

2. As a general comment, after authors' description of single symptoms included among "sensory and autonomic" I would rather define them as "non motor" since insomnia is questionable as sensory or autonomic dysfunction.

***Response****: This has been redefined as requested in the Methods section on Lines 316 and 321.*

**NOTE:** Please note that we have updated reference number 40.
