# Supplementary material for: A phase II open label clinical study of the safety, tolerability and efficacy of ILB® for Amyotrophic Lateral Sclerosis
Source: PLoS One. 2022 May 25;17(5):e0267183. doi: 10.1371/journal.pone.0267183 (PMC9132272; doi:10.1371/journal.pone.0267183)
Supplement: Attachment — Submitted filename: Revision_PONE-D-21-04468_R2_reviewer.docx [file pone.0267183.s008.docx]

Ann Logan et al.

My concerns have been adequately addressed and the more substantial concerns by the other reviewers have also been met.
