# Supplementary material for: A phase II open label clinical study of the safety, tolerability and efficacy of ILB® for Amyotrophic Lateral Sclerosis
Source: PLoS One. 2022 May 25;17(5):e0267183. doi: 10.1371/journal.pone.0267183 (PMC9132272; doi:10.1371/journal.pone.0267183)
Supplement: Attachment — Submitted filename: Response to Reviewer V4.docx [file pone.0267183.s009.docx]

*Ann Logan et al.* A phase II open label clinical study of the safety, tolerability and efficacy of ILB^®^ for Amyotrophic Lateral Sclerosis

**Response to Editor and Reviewer #2**

**Editor**

We have appreciated the detailed and helpful comments of the reviewers that we have considered carefully throughout the revision process, but we were surprised to receive a fourth round of comments after such a protracted review period. We understand that it is usually desirable to achieve ‘sign off’ from all reviewers but perceive that this may be unobtainable with Reviewer #2. As further comment, some of the authors are very experienced long-standing researchers including one who is acknowledged as amongst the World’s Most Highly Cited Authors and an Editor-in-Chief of a leading pharmacology journal, yet the precedence of a fourth round of comments is new to all of the authors. Nevertheless, we are happy to hear that we have indeed addressed all of their previous concerns relayed to us in our revised manuscript (version 3) and here we once again try to address the further concerns of Reviewer #2.

We are dismayed by the misleading comments by Reviewer #2 who seems to disregard (or be unaware of) the current clinical trial design and reporting recommendations accepted by your journal (see details below). Although we believe that it is unnecessary, please let us know if you wish us to include the extra paragraph on methodology set out below (yellow highlighted section). With the offer to insert the extra paragraph if considered necessary, we hope the Editor is now in a position to make a final judgement on our submission.

**Reviewer #2**

1. Thank you for your response to my previous comments. I'm unclear why the sample size is what it is still. Why 15? Is it an absence of any SAEs or dropouts?

***Response****: The protocol planned for 15 patients to be recruited into the clinical trial. This report describes the accumulated data from the first 13 patients recruited into the clinical trial, that was halted early due to the drug’s confirmed safety profile as there were no SAE or dropouts during the study (as clearly described in the clinical trial results and in the paper, see Tables).*

*This trial is a phase II exploratory trial (see protocol at* [*https://www.clinicaltrialsregister.eu/ctr-search/trial/2017-005065-47/SE#A*](https://www.clinicaltrialsregister.eu/ctr-search/trial/2017-005065-47/SE#A)*). The main aims were to explore the safety of the drug in the target population (phase I data already available in a healthy population) and* ***identify*** *potential for therapeutic efficacy of this new treatment for ALS. The results of this exploratory trial will contribute to the informed design of a phase III trial if/when applicable.*

*Presumably being well versed in clinical trial methodology, Reviewer #2 is probably well aware that, due to the lack of information on effect sizes, formal power and sample size calculations for phase II trials are difficult and there is no consensus regarding the best methodology. See:*

*Nigel Stallard. Optimal sample sizes for phase II clinical trials and pilot studies. Statist. Med. 2012, 31 1031–1042. DOI: 10.1002/sim.4357*

*Steven A. Julious. Sample size of 12 per group rule of thumb for a pilot study. Pharmaceut. Statist. 2005; 4: 287–291 DOI: 10.1002/pst.185.*

*The issue is even more complex for such exploratory (or pilot studies) when the disease is a rare disease such as ALS. Hence the recommendation of the SPIRIT 2013 statement for clinical trial design:*

*An-Wen Chan et al., SPIRIT 2013 explanation and elaboration: guidance for protocols of clinical trials. BMJ Research Methods and Reporting. BMJ 2013;346:e7586 doi: 10.1136/bmj.e7586*

*This publication accepts that circumstances can dictate that sample size is not derived by statistical calculations. It was this principle that led to the acceptance of the empirically chosen n=15 by the ethics committee who approved the ALS clinical trial described. Additionally, the n=15 satisfies the recommendations of Julious (see reference above) for exploratory trials (minimum recommended n=12; paper attached). The lack of a formal power analysis and sample size calculation is clearly stated i.n our paper (lines 347-348) as recommended by the SPIRIT 2013 statement.*

*In our submitted paper we endeavour to describe the trial (from design to completion) with honesty and integrity without trying to embellish the findings with post-hoc power analyses of doubtful value. See:*

*Yiran Zhang et al., Post hoc power analysis: is it an informative and meaningful analysis? Zhang Y, et al. General Psychiatry 2019;32:e100069. doi:10.1136/gpsych-2019-100069*

*However, we understand that Reviewer #2 does not want to allow publication of the study without power calculations and sample size estimates. Therefore, we believe it is an editorial decision whether the following paragraph should be included at the end of our statistical analysis:*

*“The trial is a phase II exploratory trial. There was no formal sample size estimate or power calculation. The sample size was determined empirically and reflects the exploratory nature of the trial and the rarity of ALS (which is an orphan disease; Chan et al., SPIRIT 2013 statement^1^). However, at the insistence of the reviewers and editor we have explored whether the trial design would have been admissible using validated approaches to phase II clinical trial design (Jung et al^2^ and Lin et al^3^). For design assumptions in this exploration we did NOT use the actual outcome of the trial but relied on what was known in the literature about ALS trial outcomes at the time (high placebo effect and acceptable SAE rate for riluzole) which would have been a reasonable, albeit ambitious, expectation for a new drug. Fleming’s two-stage design (Jung et al^2^) was used (software made available by the University of North Carolina at Chapel Hill,* [*http://cancer.unc.edu/biostatistics/program/ivanova/FlemingsTwoStageDesign.aspx*](http://cancer.unc.edu/biostatistics/program/ivanova/FlemingsTwoStageDesign.aspx) *) with the following assumptions: the null hypothesis that the response rate is 0.3 (based on the observed placebo effect in ALS trials). The drug will be regarded as good if the desirable effect (positive response = at least no decline in ALSFRS score) is observed in at least 70% of the patients. We expect the type I error rate of 0.05 and 80% power when the positive response rate is 0.7. The results of the analysis are summarised in Table X below.*

***Table X****. Exploration of trial design and power calculations. Where n is the total number of subjects; n_1_ is the number of subjects accrued during stage 1; a_1_, if a_1_ or fewer responses are observed during stage 1, the trial is stopped early for futility; b_1_, if b_1_ or more responses are observed during stage 1, the trial is stopped early and H0 is rejected; a_2_, if a_2_ or fewer responses are observed by the end of stage two, then no further investigation of the drug is warranted; b_2_, b_2_ = a_2_ + 1, if b_2_ or more responses are observed by the end of stage two, H0 is rejected; EN_0_ is the expected sample size for the trial when response rate is 0.3; EN_1_ is the expected sample size for the trial when response rate is 0.7.*

| ***n*** | ***n*_1_** | ***a*_1_** | ***b*_1_** | ***a*_2_** | ***b*_2_** | **Type 1 Error** | **Power** | ***EN*_0_** | ***EN*_1_** | **Comment** |
| --- | --- | --- | --- | --- | --- | --- | --- | --- | --- | --- |
| 10 | 7 | 3 | 5 | 5 | 6 | 0.0498 | 0.825 | 7.2917 | 7.6807 | Minimax |
| 10 | 6 | 2 | 5 | 5 | 6 | 0.0472 | 0.8379 | 6.979 | 8.0374 | Minimax |
| 10 | 7 | 3 | 5 | 5 | 6 | 0.0498 | 0.825 | 7.2917 | 7.6807 | Minimax |
| 11 | 5 | 1 | 4 | 6 | 7 | 0.0435 | 0.8136 | 7.646 | 7.646 | Optimal |
| 13 | 5 | 2 | 5 | 6 | 7 | 0.0408 | 0.815 | 6.2852 | 10.3508 |  |
| 14 | 5 | 2 | 4 | 7 | 8 | 0.0439 | 0.8064 | 6.1907 | 7.7783 | Optimal |
| 14 | 5 | 2 | 4 | 7 | 8 | 0.0439 | 0.8064 | 6.1907 | 7.7783 | Optimal |

*From Table X above it is clear that a trial design including a total of 15 patients is adequate to test the possible efficacy of the drug. It is also clear that stopping the trial early, when 13 patients were recruited did not affect the power of the trial.*

*Using the BOP2: Bayesian Optimal Phase II Design software (available from the University of The University of Texas MD Anderson Cancer Center:* [*https://www.trialdesign.org/one-page-shell.html#BOP2*](https://www.trialdesign.org/one-page-shell.html#BOP2) *; Lin et al^3^) we have checked the validity of our design with one interim analysis allowed. The expectations for efficacy outcome were the same as described above (placebo effect: 30% positive outcome, accepted good drug effect: 70% positive outcome). Additionally, the accepted maximum rate for SAE was 5% (observed with Riluzol (Bensimon et al^4^) for the null hypothesis. The expectation was that with ILB^®^ we have no SAE (stated stopping criteria 1 patient with SAE possibly related to the drug). We also assume that the efficacy and toxicity of the drug are not related and expect a type I error rate at or below 0.05. With these assumptions and with a maximum of 13 patient enrolled, the results of the 10000 simulations are shown in Table Y below. The power of this trial would be: 0.814.*

***Table Y.*** *BOP2: Bayesian Optimal Phase II Design with Simple and Complex Endpoints*

*BOP2 PID: 960; Version: V1.4.10.0; Last Updated: 8/24/2021 (*[*https://www.trialdesign.org/one-page-shell.html#BOP2*](https://www.trialdesign.org/one-page-shell.html#BOP2)*, used on 07/12/2021).*

| **Number of patients treated** | **Stop if # response <=** | **OR # toxicity >=** |
| --- | --- | --- |
| 7 | 2 | 1 |
| 13 | 6 | 1 |

*The analysis would indicate that the empirically chosen sample size (with reasonable expectations based on existing knowledge at the time) would have been deemed adequate for a phase II trial. These estimates were produced at the request of reviewers and the editor and are not meant to be a post hoc power analysis based on the outcome of the trial (Zhang et al^5^).”*

***References***

*^1^An-Wen Chan et al., SPIRIT 2013 explanation and elaboration: guidance for protocols of clinical trials. BMJ Research Methods and Reporting. BMJ 2013;346:e7586 doi: 10.1136/bmj.e7586*

*^2^* *Sin-Ho Jung et al., Admissible two-stage designs for phase II cancer clinical trials. Statist. Med. 2004; 23:561–569 (DOI: 10.1002/sim.1600)*

*^3^Ruitao Lin et al., BOIN12: Bayesian Optimal Interval Phase I/II Trial Design for Utility-Based Dose Finding in Immunotherapy and Targeted Therapies. JCO Precis Oncol 4:1393-1402*

*^4^Gilbert Bensimon et al., Riluzole treatment, survival and diagnostic criteria in Parkinson plus disorders: The NNIPPS Study. Brain 2009: 132; 156–171. doi:10.1093/brain/awn291*

*^5^Yiran Zhang et al., Post hoc power analysis: is it an informative and meaningful analysis? Zhang Y, et al. General Psychiatry 2019;32:e100069. doi:10.1136/gpsych-2019-100069*

2. With what power is one wanting to rule out a given rate of SAEs? Where is this stated a priori in the protocol?

***Response****: It is stated in the clinical trial protocol and in the paper that no power calculations were performed. For additional detail see above.*

3. An aspiration is what one is looking for - it is not a measure of outcome - so this still remains missing.

***Response****: The ‘aspiration’ of the phase II exploratory trial is to ‘explore’, rather than set expectations out of thin air. The reviewer is setting expectations that are fit for a phase III trial and does not seem to be fully aware of the current clinical trial design and reporting recommendations accepted by the journal (see SPIRIT 2013 statement).*

4. There is still on (*sic:* no?) statistical hypothesis.

***Response****: That is correct. There is no statistical hypothesis because there is no need for it in a phase II trial (see above).*

5. Dynamite plots are well known as (sic: for?) conveying less information that (sic: than?) in (sic: is the in necessary?) a table not least as (sic: because the?) spread of data is hidden. Why not give actual data? And why not at the very least give n, mu and sd. This is a commonly accepted minimum, even if one does not accept the consensus of statisticians, presented in <https://simplystatistics.org/2019/02/21/dynamite-plots-must-die/> . If I find Fig 6 unclear and simply a forest of bars that does not provide the evidence to support the text, other readers will too. The issue is to provide the evidence not aesthetic beauty.

***Response****:*

5.1. *The comment above is written in poor English and lacks clarity. It certainly falls short of the expectations of a quality peer review.*
5.2. *The link (given as a reference regarding the quality of dynamite plots) is from a 2019 twitter post of Prof Rafael Irizarry. The link does not lead to an active page (Error message: “404 File not found, The site configured at this address does not contain the requested file” checked on the 13/12/2021). Perhaps in peer reviews it would be better to use references that the reviewer has actually read – a catchy title from a famous statistician may not necessarily mean “consensus of statisticians”. Journal articles or book chapters may be a better source for scientific guidance to authors than unchecked links lifted from old twitter posts.*
5.3. *It is only Figures 4 and 5 that contain cumulative data from the 13 patients. Both graphs give the mean and standard deviation of the group (which is clearly labelled on the graphs) as expected by the reviewer. The n number is 13 as stated several times in the paper. The actual data included in these figures are given in Tables 7 and 8 for each individual patient. So, the “why not give actual data?” and “why not at the very least give n, mu and sd” can only refer to Figure 6.*
5.4. *While it is great to hear that Figure 6 was aesthetically pleasing, we must inform the Reviewer and Editor that this* ***figure was removed from the last submitted revised version*** *of the paper and replaced by Table 7 containing the actual numerical data.*
5.5. *It may be worth noting that Figure 6 was NOT a dynamite plot anyway. Figure 6 was “Comparison of ALSFRS-R and Norris scores for individual patients at Visit 2 (V2 at Day 1; prior to first ILB® injection) and Visit 7 (V7 at Day 36; 7 days after last ILB® injection)”. So, it was the actual individual scores of patients measured at the two identified time points. There was nothing hidden, the graph contained the actual data from the patients rather than cumulative means. This was made clear in title and legend of the figure. While one would hope that an average reader would read at least the title of the figure before bemoaning the lack of ‘evidence’, the figure was replaced by Table 7 that is ‘less aesthetically pleasing’ but is clear even to the most superficial reader.*
