# Supplementary material for: A phase II open label clinical study of the safety, tolerability and efficacy of ILB® for Amyotrophic Lateral Sclerosis
Source: PLoS One. 2022 May 25;17(5):e0267183. doi: 10.1371/journal.pone.0267183 (PMC9132272; doi:10.1371/journal.pone.0267183)
Supplement: Attachment — Submitted filename: Response to reviewer .docx [file pone.0267183.s010.docx]

*Ann Logan et al.* A phase II open label clinical study of the safety, tolerability and efficacy of ILB^®^ for Amyotrophic Lateral Sclerosis

**Response to Editor**

We have appreciated the detailed and helpful comments of the editor and reviewers that we have considered carefully throughout the revision process.

We have carefully evaluated the most recent (fifth round) comments of the Editor and have added a new S2 Appendix and a short section in the Methods (Statistical analysis, P18-19, Lines 345-350) regarding the validity of the patient number evaluated as requested.
