# Supplementary material for: A phase II open label clinical study of the safety, tolerability and efficacy of ILB® for Amyotrophic Lateral Sclerosis
Source: PLoS One. 2022 May 25;17(5):e0267183. doi: 10.1371/journal.pone.0267183 (PMC9132272; doi:10.1371/journal.pone.0267183)
Supplement: Attachment — Submitted filename: Response to reviewer .docx [file pone.0267183.s011.docx]

*Ann Logan et al.* A phase II open label clinical study of the safety, tolerability and efficacy of ILB^®^ for Amyotrophic Lateral Sclerosis

**Response to the Editor**

*We have made 4 changes to the manuscript (highlighted in red in the tracked manuscript.*

1. *On Page 1 Line 7-9: At his request we have removed Bernardo Ropero from the author list.*
2. *On Page 2 Line 27 we have corrected the email address of the corresponding author.*
3. *On Page 18 Line 348 we have corrected the supplementary appendix notation.*
4. *On Page 36 Line 675 we have removed reference to the funding for the study.*
5. *On Page 46 Lines 915-917 we have corrected the list of supplementary material.*

**Response to Reviewer #2**

Reviewer #2: I am curious about supplement 2 - where did the numbers for efficacy etc come from - were they decided a priori? I can find no mention of 70% in the protocol - please can you point me to the a priori decisions on minimum acceptable efficacy and maximal acceptable toxicity

*We have carefully evaluated the most recent comments of the Reviewer #2 and have added a new section in the S2 Appendix (Lines 18-42, Lines 74-76 and 4 new references on Lines 104-110) regarding the chosen response rates and toxicity as requested.*
